# Bacterial-Nanocellulose-Based Biointerfaces and Biomimetic Constructs for Blood-Contacting Medical Applications

**DOI:** 10.1021/acsmaterialsau.3c00021

**Published:** 2023-06-27

**Authors:** Erin L. Roberts, Sorosh Abdollahi, Fereshteh Oustadi, Emma D. Stephens, Maryam Badv

**Affiliations:** † Department of Biomedical Engineering, Schulich School of Engineering, University of Calgary, 2500 University Drive NW, Calgary, Alberta, Canada, T2N 1N4; ‡ Libin Cardiovascular Institute, University of Calgary, 3330 Hospital Drive NW, Calgary, Alberta, Canada, T2N 4N1

**Keywords:** bacterial nanocellulose, hemocompatible, biointerfaces, hemostatic biomaterials, anticoagulant, biomaterials, vascular grafts, blood-contacting biomaterials, biofunctional

## Abstract

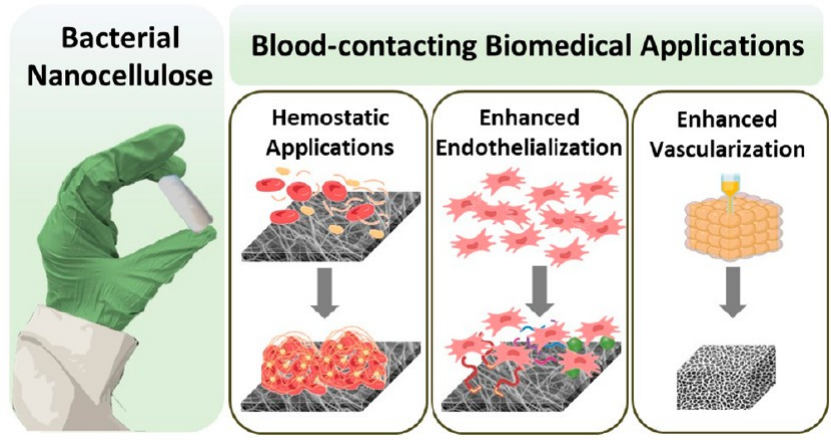

Understanding the interaction between biomaterials and blood is critical in the design of novel biomaterials for use in biomedical applications. Depending on the application, biomaterials can be designed to promote hemostasis, slow or stop bleeding in an internal or external wound, or prevent thrombosis for use in permanent or temporary medical implants. Bacterial nanocellulose (BNC) is a natural, biocompatible biopolymer that has recently gained interest for its potential use in blood-contacting biomedical applications (e.g., artificial vascular grafts), due to its high porosity, shapeability, and tissue-like properties. To promote hemostasis, BNC has been modified through oxidation or functionalization with various peptides, proteins, polysaccharides, and minerals that interact with the coagulation cascade. For use as an artificial vascular graft or to promote vascularization, BNC has been extensively researched, with studies investigating different modification techniques to enhance endothelialization such as functionalizing with adhesion peptides or extracellular matrix (ECM) proteins as well as tuning the structural properties of BNC such as surface roughness, pore size, and fiber size. While BNC inherently exhibits comparable mechanical characteristics to endogenous blood vessels, these mechanical properties can be enhanced through chemical functionalization or through altering the fabrication method. In this review, we provide a comprehensive overview of the various modification techniques that have been implemented to enhance the suitability of BNC for blood-contacting biomedical applications and different testing techniques that can be applied to evaluate their performance. Initially, we focused on the modification techniques that have been applied to BNC for hemostatic applications. Subsequently, we outline the different methods used for the production of BNC-based artificial vascular grafts and to generate vasculature in tissue engineered constructs. This sequential organization enables a clear and concise discussion of the various modifications of BNC for different blood-contacting biomedical applications and highlights the diverse and versatile nature of BNC as a natural biomaterial.

## 1 Introduction

Blood-contacting medical devices and implants are in high demand due to the high prevalence of cardiovascular and other diseases as well as trauma. Cardiovascular diseases cause approximately 30% of all deaths worldwide,^1^ while 40% of all deaths associated with trauma can be attributed to hemorrhage;^2,3^ therefore, it is crucial to explore new treatment options and biointerfaces to effectively address these complications. In this regard, novel engineered biomaterials have gained significant attention as a promising approach to improve outcomes for patients with cardiovascular diseases and bleeding complications and decrease mortality rates. Researchers are constantly engaged in the pursuit of developing novel biomaterials by exploring various polymers with the ultimate goal of enhancing patient outcomes and efficiently addressing complications associated with blood-contacting medical devices. Polymers that are commonly used for blood-contacting medical devices include synthetic polymers such as polyethylene glycol (PEG) and polyethylene terephthalate (PET) and natural polymers such as gelatin, fibrinogen, chitosan, and alginate.^4^ Among the various natural polymers, BNC has gained considerable attention as a versatile natural polymer with diverse applications. Depending on the modifications applied, BNC can be used to inhibit thrombosis, making it suitable for use in cardiovascular implants^5−8^ and vascularization of tissue constructs,^9−12^ or to promote thrombosis making it effective for treating life-threatening internal or external wounds that require hemostasis.^13−16^

**Figure 1:**
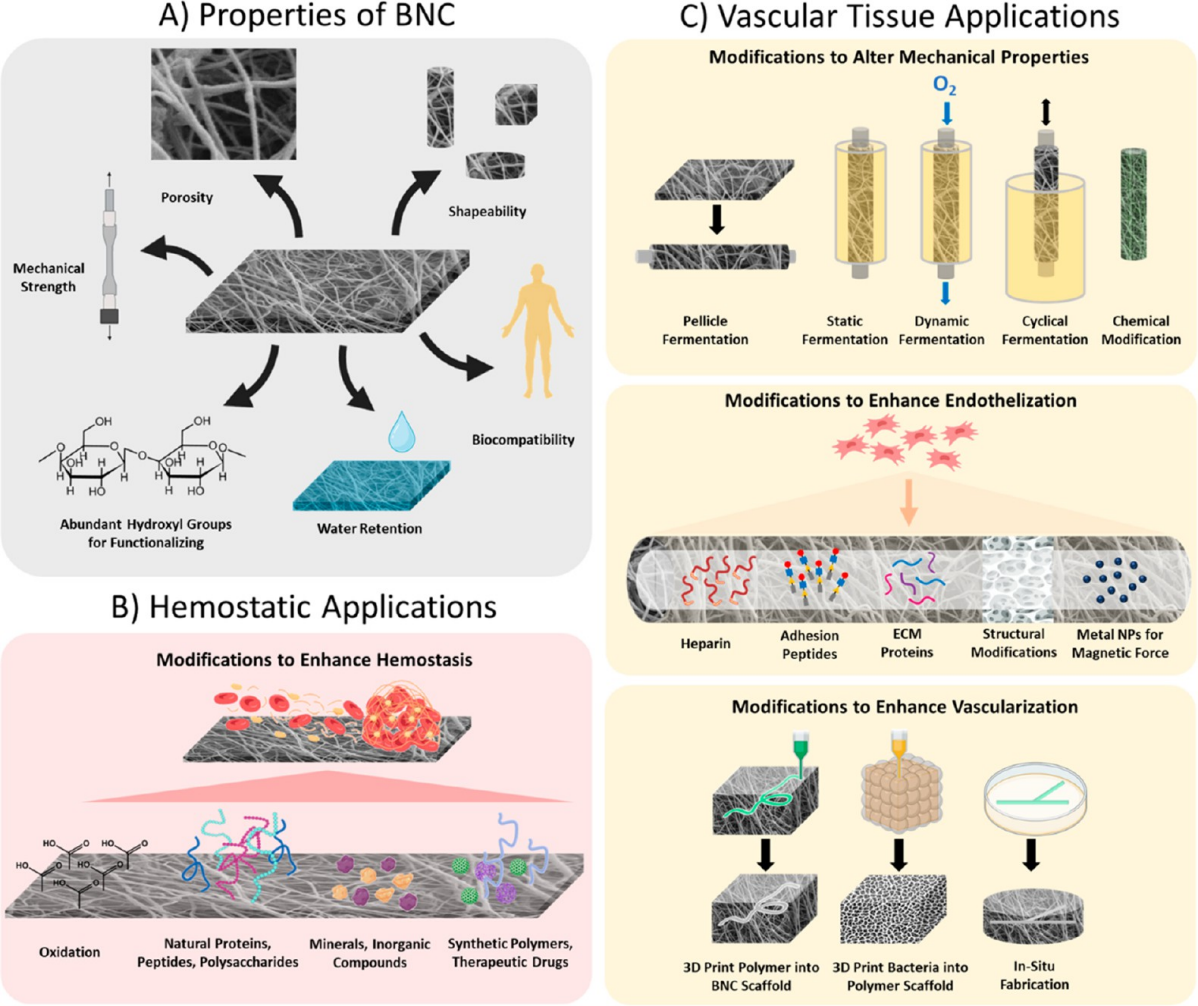
(A) Different unique properties of bacterial nanocellulose (BNC). (B) Modifications that have been applied to BNC for hemostatic applications. (C) Modifications that have been applied to BNC for vascular tissue applications.

BNC is a natural biomaterial, produced by several different microorganisms, consisting of d-glucose residues connected by glycosidic bonds.^17^ The abundance of hydroxyl groups in the structure of BNC, along with its highly porous nature, allows for cross-linking with other components to tune its chemical and physical properties and to enhance its biofunctionality. BNC also has many tissue-like properties, such as a highly porous fibrous network similar to the ECM, as well as similar mechanical properties, with respect to tensile strength and elastic modulus. In addition, BNC inherently possesses several desirable properties for biomedical applications, such as biocompatibility, shapeability, and water retention capabilities (Figure 1A).^18^

Moreover, the BNC can be modified to serve as a hemostatic dressing, facilitating blood clotting. Conventional methods to control bleeding involve the use of gauzes, adhesive tapes, and zippers that use physical forces to close the wound and halt bleeding; however, these methods have shown limited efficacy in cases of severe bleeding and do not promote wound healing.^2^ Recent research has been focused on the use of biomaterials that can actively initiate blood clotting by triggering the coagulation cascade, typically by interacting with different coagulation factors. The coagulation cascade consists of two stages: primary hemostasis where platelets form a plug at the injury site and secondary hemostasis where coagulation factors are released and aid in blood clot adherence.^4^ Secondary hemostasis has two different pathways: intrinsic and extrinsic pathways. The intrinsic pathway is triggered from negative charges present on the damaged tissue activating Factor XII, and the extrinsic pathway is triggered by tissue factors from the damaged vasculature. Both the intrinsic and extrinsic pathways merge to activate Factor X, facilitating the formation of fibrin and thrombin to reinforce the platelet plug. This process occurs rapidly, with these events occurring within seconds or minutes after onset of injury.^4,19^ Due to the activation of multiple factors in the coagulation cascade, the investigation of biomaterials capable of interacting with one or several of these factors is being researched for their potential application as hemostatic biomaterials. Several synthetic and natural polymers such as gelatin,^20^ fibrin,^21^ and PEG^22^ have been researched for hemostatic applications.^23^ Among these biomaterials, BNC stands out as a promising candidate for hemostatic applications due to its superior biocompatibility (compared to synthetic polymers, other natural polymers, as well as plant derived cellulose^24−26^), mechanical properties, and high absorption properties. BNC has been engineered to incorporate hemostatic properties, through modifications by oxidation or functionalization with proteins, peptides, polysaccharides, minerals, and other inorganic compounds (Figure 1B). BNC has also been modified to incorporate antibacterial and biomimetic properties, rendering it a versatile choice for hemostatic applications. In contrast to the application of BNC as a hemostatic dressing, BNC has been investigated for use as artificial vascular grafts, which are widely used to treat conditions such as stenosis or occlusion of blood vessels and arteries. These disorders arise due to atherosclerosis, a chronic inflammatory disorder that results in thickening of the arterial wall and obstruction of the blood flow. In many cases, intervention is necessary to open occluded arteries, often through bypass surgery, where autologous vessels are used as a standard of care. Autologous grafts are where a vessel is harvested from a different part of the body and transplanted to the diseased or damaged area. However, this approach has several disadvantages such as invasiveness, the potential for poor graft quality, or inadequate size.^7^ Due to pre-existing vascular diseases or previous surgeries, over 30% of patients do not possess functional autologous vessels, leading to a shortage that has necessitated the development and adoption of synthetic graft substitutes.^27,28^ Expanded polytetrafluoroethylene (ePTFE) and PET are the commonly used materials for vascular grafts.^29,30^ While these synthetic materials have demonstrated successful long-term outcomes in larger and medium diameter arteries, complications with thrombosis and intimal hyperplasia (the process in which the tunica intima thickens due to an overgrowth of smooth muscle cells (SMCs)) have been observed with smaller vessel sizes (<6 mm).^28,31^ Due to these limitations, the development of suitable artificial grafts that mimic the biological structure and functionality of natural vessels and promote regeneration of blood vessels following implantation is required.^28,32^

Blood vessels are made up of three layers: (1) tunica externa (outer layer) which provides structural support, (2) tunica media (middle layer) which contains elastic and muscular tissue allowing for a change in diameter of the vessel during flow, and (3) tunica intima (inner layer) which contains a smooth endothelial layer that provides the vessel’s innate anticoagulant properties.^33^ All three of these layers are important in designing and developing tissue engineered biomaterials for use as a vascular graft. Successful endothelization of the tunica intima decreases thrombogenicity,^34^ and matching the mechanical properties of the biomaterial to the tunica media and tunica externa prevents compliance mismatch which has been found to lead to intimal hyperplasia.^23^ Due to the numerous complications associated with existing synthetic vascular grafts and the infeasibility and scarcity of autologous vessels,^28,31^ BNC has emerged as a promising alternative biomaterial for vascular grafts (Figure 1C). The flexibility and shapeability of BNC have been exploited by many researchers investigating different fabrication methods, to produce tubular BNC with mechanical properties similar to native blood vessels.^35−38^ Various bioreactor configurations incorporating supports comprised of various materials as well as different gas exchange processes have been investigated for in situ fabrication of tubular BNC. Additionally, several postsynthesis modifications have been researched to produce tubular BNC from BNC pellicles. Moreover, extensive research has been conducted to increase the cytocompatibility and hemocompatibility of BNC through the addition of components such as adhesion peptides,^39−41^ ECM proteins,^32,42^ and heparin.^43^ Additionally, numerous fabrication methods have been explored to introduce vasculature into BNC tissue engineering constructs.

While there are several reviews that exist with respect to BNC production, fermentation, and their general applications in the biomedical field, this is the first known review that provides a comprehensive overview of the potential applications of BNC for blood-contacting medical applications. First, various methods to test the hemocompatibility and blood-interaction effects, cytocompatibility, antibacterial properties, and mechanical properties, of BNC are discussed. Subsequently, modifications and applications of BNC for hemostatic applications are covered. Additionally, the potential of BNC for use as a biomaterial for vascular grafts and methods used to introduce vascularization to tissue engineered constructs are highlighted.

## 2 Testing of Biomaterials for Blood-Contacting Applications

For engineered biomaterials to successfully transition from research to clinical applications, rigorous testing is required. Specifically, blood interaction testing is critical for blood-contacting applications, as it determines the extent to which blood will adhere and clot or be repelled by a biointerface. In addition, mechanical testing is important to ensure that the biomaterial’s mechanical properties are appropriate for its intended function and the properties of the surrounding tissues. Depending on the application of the biomaterial, cytocompatibility testing and antibacterial testing may also be necessary. Finally, *in vivo* testing in animal or organ-on-a-chip models is a critical step in translating the results to human treatments. By conducting comprehensive testing, researchers aim to ensure the safety and efficacy of engineered biomaterials and increase the likelihood of successful clinical applications.

### 2.1 Blood and Plasma Interaction Testing

**Figure 2:**
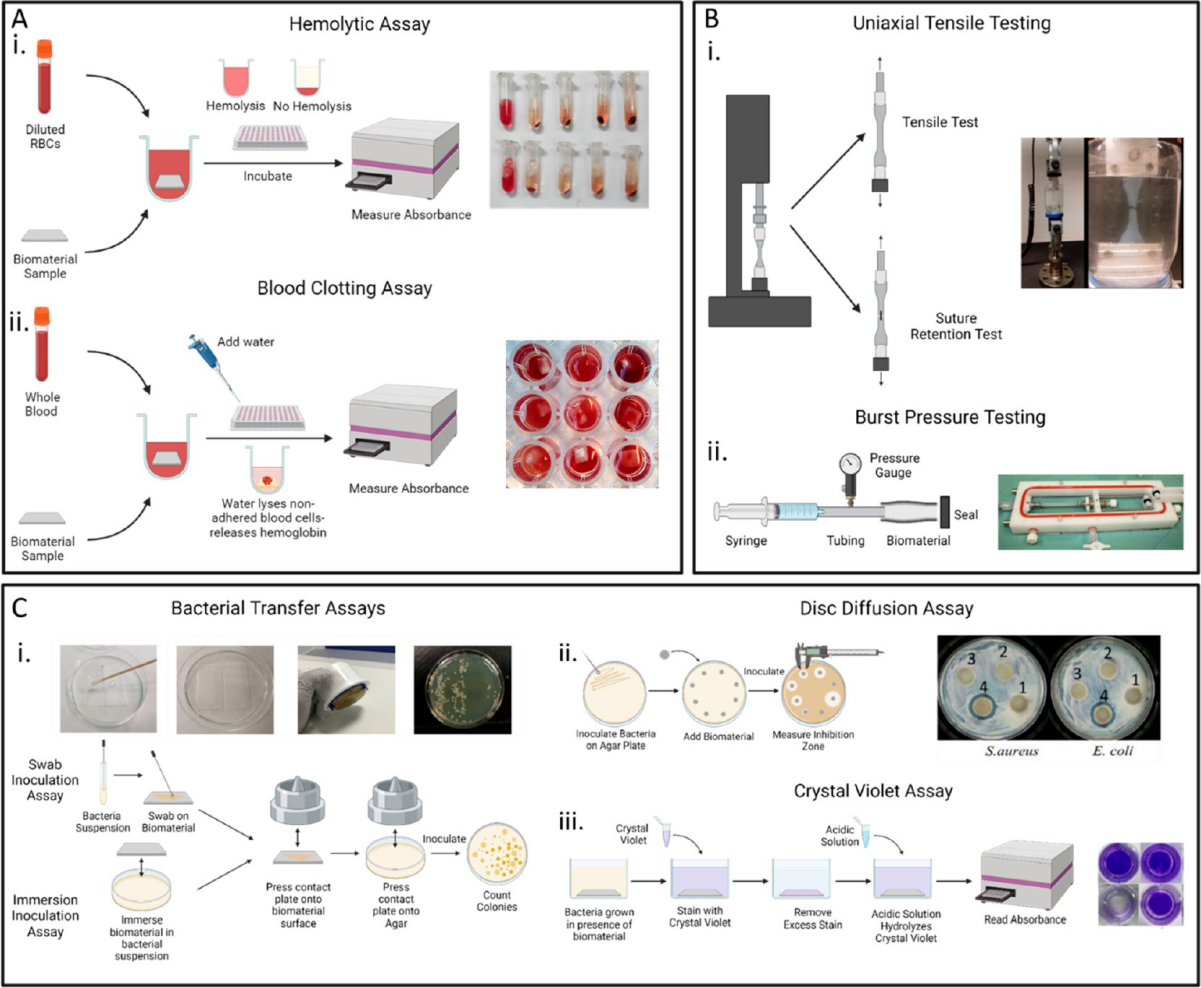
Different testing methods to assess biomaterial–blood interactions. (A) (i) Hemolysis assay to determine the extent a biomaterial lyses red blood cells. Image reprinted with permission from ref (15). Copyright 2021 Elsevier. (ii) Blood clotting assay to determine the thrombogenicity of a biomaterial. (B) (i) Uniaxial tensile testing to test the tensile strength as well as the suture retention strength of a biomaterial. Image reprinted with permission from ref (170). Copyright 2021 Elsevier. (ii) Burst pressure testing specifically important for testing the strength of vascular grafts. Image reprinted with permission from ref (69). Copyright 2014 Elsevier. (C) (i) Bacterial transfer assays including the swab inoculation assay and the immersion inoculation assay to test bacterial adhesion and transmittance by a biomaterial. Image reprinted with permission under a Creative Commons Attribution 4.0 International License from ref (91). Copyright 2021 Springer Nature. (ii) Disc diffusion assay to determine the antibacterial properties of a biomaterial. Image reprinted with permission from ref (118). Copyright 2021 from American Chemical Society. (iii) Crystal violet assay to determine biofilm growth and formation on a biomaterial. Image reprinted with permission from ref (129). Copyright 2022 Elsevier. Schematics made using Biorender.com.

To assess the blood compatibility of a biomaterial, several different *in vitro* tests can be performed (Figure 2A). A commonly performed test to ensure the hemocompatibility of a biomaterial is a hemolysis test. This test aims to determine whether the biomaterial damages red blood cells, making it a critical evaluation of the biomaterial’s safety for blood-contacting applications. The standard procedure involves exposing a dilute suspension of red blood cells to a biomaterial and measuring the absorbance using a plate reader after an incubation period. Typically, saline is used as a negative control and a surfactant is used as a positive control.^44^ The hemolysis percentage can be calculated using eq 1, where OD is the optical density.^4^ A hemolysis rate less than 2% is considered appropriate for a blood-contacting medical device.^45,46^
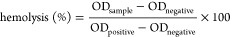
1

BNC has generally shown a low hemolysis percentage in hemocompatibility testing;^47,48^ however, various modifications to the BNC surface as well as the drying method used can affect this. Therefore, testing of the property for a given BNC membrane is required.^49^

In addition to the hemolysis test, further blood interaction testing is typically conducted to determine if a biomaterial will activate the coagulation cascade, leading to blood clotting on the biointerface. Whole blood and plasma clotting assays are among the most commonly used assays to evaluate the hemocompatibility of biointerfaces. These assays typically initiate clotting by recalcifying citrated plasma/blood, and the clotting times are recorded using various methods, including visual and optical measurements.^50^ These tests are considered an ideal screening test to determine total coagulation, as all plasma coagulation factors (excluding factor IV) are assessed in this test.^51^ In addition, factor-depleted plasma tests can be conducted to determine the pathway involved in surface-initiated plasma clotting assays by depleting specific factors involved in contact or tissue factor pathways and then initiating the clotting assay by recalcifying the plasma.^52^

There are several ways to perform optical density measurements to assess thrombosis in a biomaterial. One such method involves inducing thrombosis by adding blood or plasma on top of a biomaterial. At different time increments, the blood is diluted with distilled water, causing any blood cells that were not adhered to a clot to lyse and release hemoglobin. The absorbance of the released hemoglobin is then measured with a plate reader.^4,15^ The blood clotting index (BCI) is often reported in studies, which is calculated using eq 2, where *A* is the absorbance of blood after exposure to the biomaterial and *B* is the absorbance of citrated blood. The lower BCI indicates higher blood coagulation.^20^
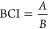
2A simple tube method is also commonly used to assess clot formation on a biomaterial. In this method, a biomaterial is placed in a tube, and blood or plasma is added on top. The tube is then tilted at different time intervals until the blood forms a dense clot and stops flowing. For blood-contacting applications such as vascular grafts where it is critical to prevent thrombosis, clotting times of over 40 min are typically considered appropriate. However, this highly depends on the type of clotting assay, sample size, and experimental setup used for measuring the clotting time.^47^ For blood-contacting applications in which hemostatic properties are desired, the faster the clotting time, the better. The clotting time is typically compared to that of a control, such as a commonly used hemostatic material, to compare the effectiveness. Clot formation can be determined by measuring the mass of the biomaterial before and after incubation to calculate the mass of the clot adhered to the biomaterial.^53^ An advantage of using plasma instead of whole blood is that this assesses the coagulation cascade without the additional components of blood cells and platelets.^54^ Specifically, two types of plasma testing commonly performed to test the extrinsic and intrinsic coagulation pathways independently are the prothrombin time (PT) and activated partial thromboplastin time (aPTT).^55^ PT is a measurement used to determine the effect of biomaterial on the extrinsic pathway of the coagulation cascade. In this assay, thromboplastin and calcium are added to plasma and the clotting time is measured. In the activated partial thromboplastin time, a measurement of the intrinsic pathway, an activator, such as kaolin or cephalin, is added to plasma and the time to clot is measured.^56^

Clot formation on biointerfaces is a complex process that involves various integrated pathways including the adhesion and activation of platelets, protein adsorption, and thrombin generation. Among these events, the adsorption of platelets and plasma proteins, such as fibrinogen, is considered the crucial initiating step.^57^ Therefore, the assessment of platelet and blood/plasma protein adhesion on biomaterials is crucial to evaluate their blood compatibility.^55^ Platelet adhesion tests involve exposing the biomaterial to extracted platelets for a specified period of time and then washing the biomaterial to remove any unadhered platelets. To quantify the adhered platelets on the biomaterial, they are lysed, typically using Triton X-100, and the lactate dehydrogenase (LDH) activity is measured using absorbance.^4^ Additionally, instead of lysing the platelets and measuring the LDH activity, the adhered platelets can be directly visualized by using scanning electron microscopy or fluorescence microscopy where activated platelets are labeled and visulized.^54,58,59^ To assess protein absorption to biomaterials, a common test to perform is to soak the biomaterial in bovine serum albumin (BSA) solution, collect the supernatant, and measure the absorbance of the supernatant before and after exposure to the biomaterial. The protein adhesion can then be quantified using eq 3, where *w* is the weight of the swollen biomaterial, *C*_0_ and *C*_a_ are the initial and final BSA concentrations, and *V* is the volume of BSA solution added.^4^
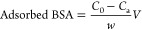
3A higher value of BSA indicates that a higher amount of protein has been adsorbed onto the biomaterial. For the application of BNC for a hemostatic material, a higher adsorption would correlate with the ability of the material to adsorb higher concentrations of blood proteins. For the application of BNC for a vascular graft, a low adsorbed BSA is required as adsorbed protein can initiate clotting.^4^

### 2.2 Mechanical Testing

The mechanical properties of a biomaterial are fundamental to the integration, functionality, and overall success and efficacy of the biomaterial for the given application. Opting for a material that exhibits similar mechanical behaviors to the native tissue ensures the implants’ long-term stability, mitigating complications associated with compliance mismatch, thereby minimizing the probability of failure.^37^ To assess the mechanical properties of BNC-based biomaterials, a uniaxial tension test can be used to determine a variety of material specifications including yield strength, Young’s modulus, ultimate tensile strength, and percent elongation (Figure 2B). Selected culturing and processing techniques, especially drying methods, significantly impact the mechanical properties and behavior of BNC. For this reason, mechanical analysis of key properties is a key component of any biomaterial development using BNC. To perform the test, a specimen is loaded into a uniaxial testing apparatus by clamping two ends into machine jogs. The cross-sectional area of the sample and the distance between the jogs are measured, and that distance is taken as the original length, *l*_0_. A force with a certain rate is applied to the biomaterial, and the change in length is measured.^60^

The yield strength (or yield stress) of a material is the stress at which elastic deformation ends and plastic deformation begins. If the yield stress is exceeded, permanent damage to the biomaterial occurs, and the probability of failure is increased. On a stress–strain curve, the yield strength can be identified as the end-point of the linear region beginning at the graphical origin, which is the elastic region. If that point is unclear, the yield strength may also be calculated using a 0.2% offset approximation, involving creating a line of the same slope as the elastic region with its origin at 0.2% strain, and determining where the approximation crosses the graph as the yield strength.^61^

One of the most common measurements used to compare biomaterials is the Young’s modulus. The Young’s modulus describes a material’s behavior during elastic deformation, which is a phase in which deformation is nonpermanent. Graphically, the Young’s modulus can be determined by taking the slope of the elastic region of a stress–strain graph, which is the region existing to the left of the yield stress.^62,63^ While the Young’s modulus of a hemostatic wound dressing is not critical to its performance, the proximity of the Young’s modulus in a vascular graft to that of the native artery is critical to its performance. A Young’s modulus that is inconsistent with that of the surrounding vasculature indicates a discrepancy in the elasticity between the graft and vasculature, which in turn creates a shear stress change within the vessel. It has been concluded through several studies that shear stress differentials in blood vessels increase the patient’s risk for developing atherosclerotic plaque.^23,64,65^ Another specification of a material that can be determined using uniaxial tensile testing is the ultimate tensile strength, which is characterized by the stress at rupture. This parameter is generally less than the yield strength in polymeric biomaterials. This phenomenon can be explained by the gradual plastic deformation and microscopic material failures prior to bulk material failure.^61^

Percent elongation (or ductility) of a material can be determined at different stages of deformation and is a useful parameter to understand a material’s behavior under different conditions. The percent elongation is given by eq 5, where *l*_0_ is the original length and *l* is the length after deformation. The specific stress value on a stress–strain graph of a biomaterial allows it to be directly compared to native tissue at the same stress.^61^
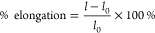
4Other mechanical tests performed on BNC-based biomaterials are selected based on their relevance to a specific application. In several studies to examine the potential of BNC as a biomaterial, the suture retention strength is often characterized. Typically, common sutures made of nylon and Dacron are used to attach two pieces of a biomaterial.^5,66^ The biomaterial is then loaded into a uniaxial testing apparatus, and a tensile force is applied (Figure 2B). The force at which the sutures fail is measured and taken as the suture retention strength.^67,68^

Burst pressure is commonly examined in tubular biomaterials as a measure of circumferential tensile strength.^37^ Specifically, for use as artificial vascular grafts, this test is important to ensure that the material will be able to withstand physiological pressures within blood vessels. To determine a material’s burst pressure, tubing and a pressure gauge are attached to one end of the material’s lumen while the other end is sealed. Fluids are added to the vessel until it fails, and the internal pressure is recorded as the burst pressure.^69^ When determining the viability of a biomaterial as a vascular graft, the biomaterials’ circumferential strength should be directly compared to that of native tissues.^67,68^ In vascular graft applications, mechanical discrepancies between synthetic and native materials can create a pressure differential that can lead to the premature failure of the graft. Mechanical compliance, the inverse of stiffness, can also be used to compare the behavior of synthetic vessels to that of native tissue under regular physiological strain. A high compliance means that the biomaterial is displaced to a high degree when a load is applied. To establish compliance, samples are fixed in a chamber and subjected to a steady flow of pure water or more complex biological fluids such as blood or plasma. A pulse is then sent through the fluid, and the diameter of the vessel at the moment of the pulse (systolic diameter, *D*_systolic_) is measured and compared to the diastolic diameter (*D*_diastolic_). Based on the vessel pressure under systolic (*P*_systolic_) and diastolic (*P*_diastolic_) conditions and the systolic and diastolic diameters, material compliance can be calculated using eq 5.^5,64^
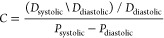
5

### 2.3 Antibacterial Testing

Two common approaches can be used to impart antibacterial properties to BNC biointerfaces: active and passive. In the active approach, antibacterial agents such as antibiotics,^70−72^ metal nanoparticles,^73−75^ carbon nanoparticles,^76,77^ nanosilicates,^78,79^ or organic components such as chitosan^16,80,81^ are loaded within the porous structure of BNC through physical entanglement^71,74,76^ or chemical immobilization.^72,73,77^ These agents can be released at a controlled rate or could be immobilized on the surface and provide continuous antibacterial activity at the implant site. In contrast, in the passive approach, BNC membranes are modified with a super-repellent coating and passively prevent bacterial adhesion and biofilm formation on their surfaces.^80,82−84^

To evaluate the antibacterial properties of the developed biomaterials, tests such as the disc diffusion test^85−88^ or suspension culture assays^87,89^ can be performed. The disc diffusion test involves placing the biomaterial onto agar plates inoculated with bacteria. The antibacterial activity is then assessed through the measurement of an inhibition zone, typically reported as a diameter or area of clearance or as arbitrary units which are defined as the reciprocal of the highest dilution at which the growth of the indicator pathogen is inhibited (Figure 2C). A drawback of this test is that different antibacterial agents within the biomaterial will have various diffusion rates, affecting the size of the inhibition zones. However, if a single antibacterial agent is being tested against several different bacterial strains, this limitation is avoided.^86^ Determining the antimicrobial activity of an antibacterial-loaded biomaterial is also done by using suspension culture assays. In this test, aliquots of bacterial suspensions are inoculated into each well containing a biomaterial sample or a control sample. After incubation, the optical density is measured, and the growth rate of the bacteria can be estimated.^90^

To test the antibacterial properties of super-repellant biomaterials without any antibacterial agents, transfer assays, such as immersion inoculation assays,^91,92^ touch transfer assays,^91,92^ or swab inoculation assays,^86,91^ are conducted (Figure 2C). The advantage of these tests compared to the agar diffusion and suspension culture assays is that they measure the quantity of bacteria that can attach on a biomaterial surface and subsequently be transferred to another surface.^91,92^ These tests use different methods of transferring bacteria to biomaterials, followed by using contact plates to transfer any bacteria adhered to the biomaterial to agar plates, which are then incubated, and the colonies are counted. In the immersion inoculation assay, bacteria are transferred to samples through immersion in a dilute bacterial suspension. In the touch transfer assay, a sterile velvet pad is mounted on a replica plating instrument and is submerged into a dilute bacterial suspension, placed onto another dry, sterile velvet pad to remove the extra amount of bacterial suspension, and then pressed onto the biomaterial. The swab inoculation assay transfers the bacteria to the biomaterial using a swab.^91^ The swab inoculation assay and touch transfer assay mimic real-life conditions more accurately than the immersion inoculation assay based on the method of bacterial transfer to the biomaterial.^91^

Biofilm formation can also be assessed to test the antibacterial properties of both active and passive surface coatings on biomaterials. Bacteria reproduce rapidly; therefore, when attaching to a new surface, they activate signaling pathways to colonize followed by biofilm formation.^93^ The crystal violet assay is a straightforward and high-throughput method used for tracking microbial adhesion and biofilm formation on biointerfaces. In this method, bacteria are grown in well plates containing biomaterials. Surface-bound bacteria are stained with a crystal violet solution; the excess dye is removed, and the remaining surface-associated dye is solubilized with acidic solutions. The amount of light absorbance by the resulting solution is measured to quantify the amount of biofilm present.^94−96^ For testing long-term antibacterial properties of coatings, colony forming biofilms and the Kadouri drip-fed biofilm assay can be performed. Colony forming biofilm assays are a type of biofilm assay that involves growing bacteria on a semipermeable biomaterial placed on an agar plate with nutrients transferring through a membrane. To replenish the nutrient supply, the biomaterial can be transferred to a fresh agar plate.^97^ To quantify the biofilm, the biomaterial is then transferred to another agar plate with colonies facing the fresh agar surface and incubated. The number of colonies on the new agar plate is used to determine the amount of biofilm formed on the membrane.^98^ In contrast to the previous methods, in which a batch culture is used, the Kadouri drip-fed biofilm assay uses a continuous process in which bacterial culture broth is pumped into a chamber holding the test biomaterial while waste and planktonic cells exit through another port and are pumped away. Due to the continuous replenishment of nutrients and removal of waste, the growth of bacteria can be maintained for a longer time, allowing formation of mature biofilms. Shear stress is also introduced into the system, which allows for the testing of biofilm formation under various hydrodynamic conditions. However, a disadvantage of this assay is that it is low throughput, is time-consuming, and requires specialized equipment.^99^

### 2.4 *In Vivo* Testing

*In vivo* tests are crucial to determining the clinical translational potential of biomaterials and evaluating their performance in complex biological environments. Common models to test the hemostatic properties of biomaterials include a rat liver, artery, or tail model.^62−64^ These tests involve creating an incision in the organ, followed by treatment with the biomaterial. Disease models are also sometimes tested, such as a diabetic animal model.^14^ Outputs that are commonly measured are the time to hemostasis (i.e., the time taken to stop the bleeding) as well as the volume of blood absorbed by the hemostatic biomaterial.

For most biomedical applications, including artificial vascular grafts, initial *in vivo* testing typically involves a subcutaneous implant to assess the immune reaction and degradability or absorbability of the biomaterial.^66^ Small animal models, such as rabbits, mice, and rats, are used for these experiments.^10,66,100−103^ More thorough *in vivo* testing involves surgery in which arteries are replaced with synthetic vascular grafts. Following a set period of time, the animals are sacrificed, and the vascular graft is removed and subjected to various tests, such as mechanical testing and immunohistochemistry or immunofluorescence to analyze tissue, ECM, and cell attachment on the graft. The graft can also be assessed for any signs of thrombosis.^66,104^

## 3 Bacterial Nanocellulose as a Hemostatic biomaterial

While BNC exhibits tissue-like mechanical properties and biocompatibility *in vivo*, further surface and bulk modifications to BNC-based membranes are required to improve the efficacy of this biopolymer in blood-contacting applications.^49,105^ In this section, different surface and bulk modification techniques are highlighted that alter the blood-interaction properties of BNC. These modification techniques mainly involve functionalizing BNC with procoagulant synthetic or natural polymers, macromolecules, and/or hemostatic agents. Despite the great potential of the use of BNC for hemostatic applications, there are relatively few studies that have researched modifications of BNC to enhance its hemostatic properties; therefore, this area of study holds great potential to enhance the research field of hemostatic materials.

### 3.1 Oxidized BNC

Oxidation is a modification technique that has been applied to the BNC to enhance its hemostatic properties. For example, TEMPO (2,2,6,6-tetramethylpiperidine-1-oxyl radical) mediated oxidation involves the addition of COO^–^ groups to the BNC surface through the oxidation of the abundant hydroxyl groups present in the BNC structure. While the exact mechanism by which oxidized BNC (OBNC) enhances the hemostatic properties is unknown, several hypotheses have been proposed.^75^ One hypothesis suggests that the addition of the carboxyl groups lowers the pH, which converts hemoglobin to acid hematin, releasing iron ions that combine with the carboxyl groups and promote clot formation. Additionally, the drop in pH is also believed to lead to the aggregation of platelets.^106^ Another possible mechanism involves the negatively charged surface of OBNC, which can strongly adsorb to the positively charged coagulation factors XII, XI, high molecular weight kininogen and prekallikrein, initiating the intrinsic pathway of the coagulation cascade.^107,108^ According to a study by Ryšavá et al. the primary mechanism by which OBNC promotes hemostasis is through its role in thrombin generation, with the secondary mechanisms involving the activation and adhesion of platelet fibrin formation.^109^ OBNC is physically similar to unmodified BNC in that it is hydrophilic and absorbs blood and through these mechanisms traps blood proteins and blood cells, increasing clot potential.

Queirós et al. conducted a study to compare the hemostatic properties of OBNC membranes to nonoxidized BNC membranes using two different degrees of oxidation, 4% and 7% oxidation. They found that OBNC had greater hemostatic properties and decreased the whole blood clotting time when compared to unmodified BNC. Moreover, they found that increasing the degree of oxidation increased the blood clotting time.^107^ A study by Bian et al. found similar results, with OBNC having significantly higher platelet and erythrocyte adhesion, as well as an increased whole blood clotting time and plasma recalcification time when compared to control BNC samples. Moreover, in an *in vivo* study of both a rat tail amputation model and a liver trauma model, the OBNC membrane resulted in lower blood loss than the BNC.^110^ Despite the promising results reported regarding the improved hemostatic properties of OBNC, it is crucial to consider the potential drawbacks of this chemical process. Specifically, the harshness of the process can result in the disassociation of the BNC membrane, compromising its tissue-like properties and mechanical strength. Furthermore, the chemicals used during the oxidation process are often highly toxic, increasing the risk of cytotoxicity in the resulting membranes.

### 3.2 Functionalization with Natural Proteins, Peptides, and Polysaccharides

**Figure 3:**
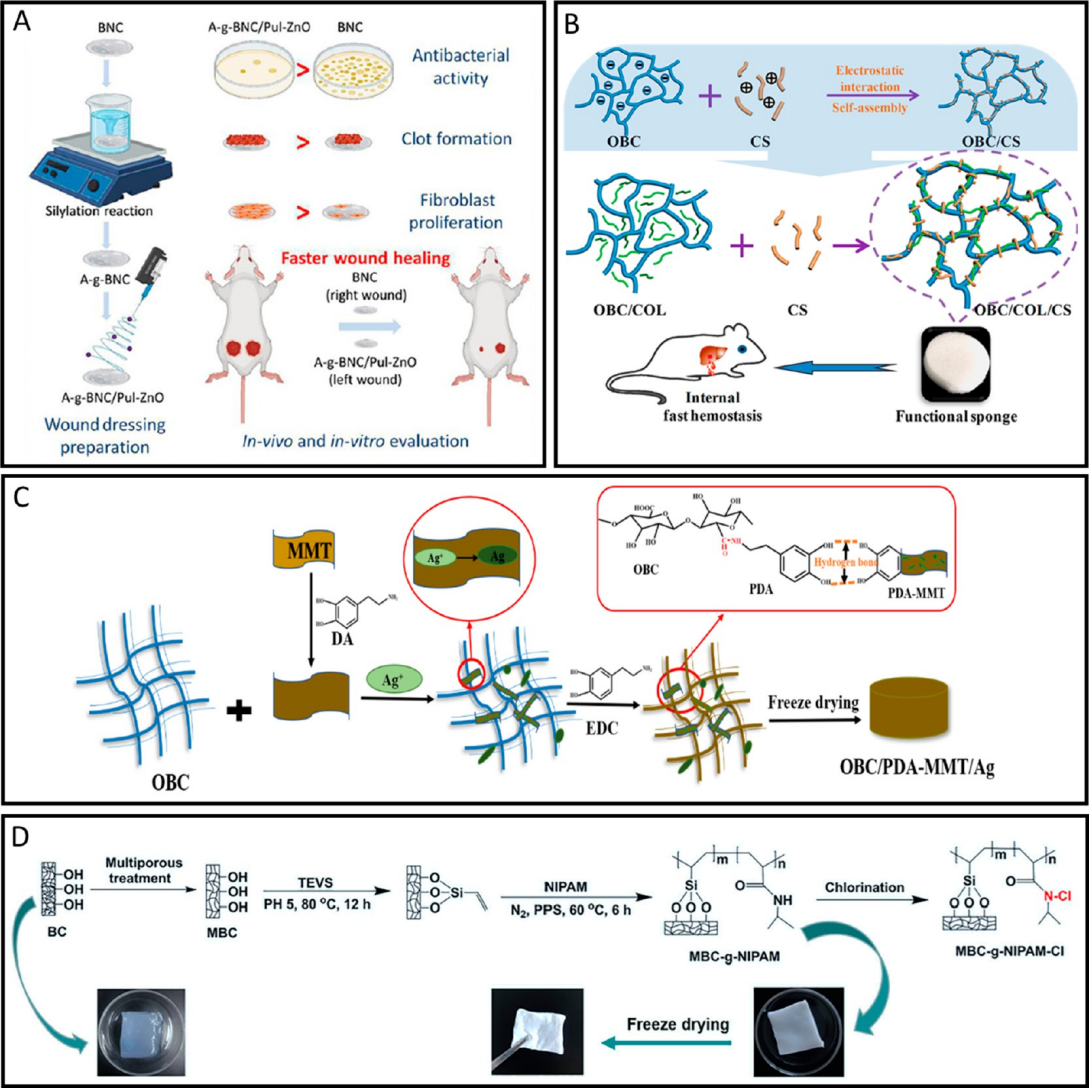
Applications of modified BNC for hemostatic membranes. (A) Oxidized BNC was functionalized with collagen and chitosan, and clot formation and fibroblast proliferation were tested *in vitro*. In addition, *in vivo* testing was performed to assess the wound healing rate of the modified and control membranes. Figure reprinted with permission from ref (16). Copyright 2020 American Chemical Society. (B) An aminoalkylsilane grafted BNC (A-g-BNC) membrane was functionalized by electrospinning pullulan (Pul)-zinc oxide nanoparticle (ZnO-NP) nanofibers, and hemostasis was evaluated *in vivo* in a mice model. Figure reprinted with permission from ref (118). Copyright 2021 American Chemical Society. (C) An OBNC membrane was functionalized with PDA, MMT, and zinc nanoparticles. Figure reproduced with permission from ref (15). Copyright 2021 Elsevier. (D) A multiporous BNC was modified with chlorinated NIPAM, and its hemostatic and antibacterial properties were investigated. Figure reprinted with permission from^58^. Copyright 2020 Elsevier.

BNC has been functionalized with different macromolecules, such as peptides, proteins, and polysaccharides, to enhance its hemostatic properties. Amino acid units, the structural component of peptides and proteins, have different electrostatic and hydrophobic properties, which enable them to interact with platelets and other components in blood though physical and chemical mechanisms to promote hemostasis.^32^ Collagen, the most abundant protein in the ECM, plays an important role in coagulation through platelet activation and facilitation of platelet adhesion and aggregation.^111^ Additionally, certain collagen types are involved in recruiting inflammatory cells to the damaged tissue.^112^ While the role of collagen in hemostasis has been extensively studied, limited studies have investigated functionalizing BNC with collagen to enhance its hemostatic properties. In one study by Yuan et al., collagen and chitosan were incorporated in an OBNC sponge (Figure 3A). This study found that the addition of collagen and chitosan significantly lowered blood loss and time to hemostasis in a rat liver trauma model as well as lowered whole blood clotting time when compared to that of control OBNC sponges. The mechanical properties of the OBNC were found to be very weak, with a tensile strength of only 12 kPa, due to damage to the BNC fibers during the oxidation process. The addition of collagen and chitosan greatly enhanced these properties, increasing the tensile strength to 129 kPa.^16^ Chitosan is a natural polysaccharide found in the outer skeleton of shellfish which has a positive charge on the amine groups on the backbone of the polysaccharide structure, allowing it to interact with negatively charged platelets, erythrocytes, and fibrinogen, promoting blood clotting and hemostasis.^68,113,114^ Chitosan is also considered antibacterial as it adheres to the cell membrane of bacteria, preventing transportation of nutrients. This study also found that the OBNC, as well as OBNC with collagen and chitosan, significantly reduced the number of bacteria in a swab inoculation assay when compared to sterile gauze. Incorporating antibacterial properties into hemostatic biomaterials is crucial as it can help minimize the risk of infection, which can impair hemostasis, prolong wound healing, and lead to potentially serious medical complications.^16^

Silk fibroin, a protein derived from the silkworm *Bombyx mori*, has been used to create hemostatic biomaterials; however, since it is usually used in combination with other biomaterials, its exact mechanism on hemostasis remains unclear.^115^ Silk fibroin can contribute to physical hemostasis through absorption of blood, swelling and forming a physical barrier.^4^ Additionally, it may activate platelets, enhance platelet aggregation, and promote blood clotting.^116^ Studies have investigated the use of electrospinning a silk fibroin coating on a BNC/chitosan dressing doped with a peptide, protamine sulfate, for inducing hemostasis in a diabetic and femoral bleeding rat model. Protamine sulfate is primarily used in surgical procedures, where it binds to heparin to create an inactive salt complex, thus reversing the anticoagulant effects of heparin.^117^ All three dressings, (1) BNC/chitosan, (2) BNC/chitosan coated with silk fibroin, and (3) BNC/chitosan/protamine sulfate coated with silk fibroin, had a significantly lower time to hemostasis, lower blood loss, and lower mortality rate than standard gauze in both rat models, with no significant differences between the three conditions.^13,14^

Along with hemostatic properties, it is also desirable to have wound healing properties in a dressing. Pullulan is a natural polysaccharide, produced by the fungus *Aureobasidium pullulans*, that has been found to be biocompatible and immunogenic. It has also been found to have wound healing properties such as increasing the rate of re-epithelization and promoting blood vessel formation.^118^ Pullulan along with zinc nanoparticles to promote antibacterial properties have been chemically grafted to BNC using an aminoalkylsilane bridge to produce a hemostatic wound dressing (Figure 3B). The BNC/pullulan/zinc nanoparticle dressing had significantly quicker blood clotting time than BNC alone; also, the addition of zinc oxide nanoparticles significantly increased the diameter of the inhibition zone in an agar spot assay and significantly decreased CFUs in a suspension bacterial assay using both *Staphylococcus aureus* (*S. aureus*) and *Escherichia coli* (*E. coli*) when comparing the BNC alone as well as BNC/pullulan. In a wound healing assay, the BNC/pullulan/zinc nanoparticle dressing had a significantly shorter time to wound closure when compared to BNC alone.^118^

Research into naturally occurring peptides, proteins, and polysaccharides as a biomaterial or a component of a biomaterial has risen greatly within the past few years, due to their biocompatibility, antibacterial properties, as well as other unique properties; therefore, this is an area of great potential for use in developing a BNC-based hemostatic dressing.

### 3.3 Minerals and Other Naturally Occurring Hemostatic Agents

Broadly defined, a mineral refers to a naturally occurring inorganic substance with an orderly internal structure and a defined chemical composition. Several minerals are considered to be biocompatible, with several applications such as dental implants, bone tissue engineering, and drug delivery.^119,120^ Kaolin and montmorillonite (MMT) are two clay minerals that have been added to BNC for their hemostatic properties. These minerals promote hemostasis primarily by activating Factor XII which initiates the intrinsic pathway of the coagulation cascade. The polar-glass-like surface of the aluminosilicate structure of these two minerals is believed to induce the “glass effect”. The “glass effect” theory suggests that coagulation occurs faster when blood comes into contact with polar-glass-like surfaces, as opposed to nonpolar plastic surfaces commonly used in medical implants.^121,122^ It has also been found that the negative charge of kaolin causes activation of XI, prekallikrien, and cofactor HWK-kininogen and MMT can activate Factors VI and VII.^121,123^ Both kaolin and MMT can also contribute to physical hemostasis through the absorption of blood.

A study by Cao et al. chemically grafted different concentrations of MMT with polydopamine (PDA) and silver nanoparticles to an OBNC sponge using 1-ethyl-3-(3-dimethylaminopropyl)carbodiimide (EDC) (Figure 3C). Polydopamine was used, as it has also been found to activate the coagulation cascade, while the silver nanoparticles provide antibacterial properties. The study found that, as the concentration of MMT increased, the blood clotting time significantly decreased *in vitro*, and blood loss was significantly decreased for all MMT concentrations when compared to gauze in an *in vivo* rat liver defect model.^15^ In addition to the hemostatic properties, the antibacterial properties of the coated OBNC membranes were investigated. OBNC membranes loaded with silver nanoparticles showed effective antimicrobial properties against *E. coli* and *S. aureus* in an agar spot assay.^15^

Several studies have evaluated the hemostatic effect of BNC membranes incorporated with kaolin through physical entanglements. It was found that BNC/chitosan doped with kaolin had a faster time to hemostasis than medical gauze in a diabetic rat femoral artery bleeding model.^14^ Additionally, it was found that the porosity of the BNC affected hemostasis, as larger pores allowed more interaction between the blood and the kaolin.^124,125^ However, as there are only physical interactions occurring between the kaolin and the BNC, there are limitations in the strength and stability of the kaolin conjugation to the BNC.

Other natural occurring components that have been used to promote coagulation include Vitamin K and phosphatidylcholine, a phospholipid.^126^ Vitamin K is a well-known anticoagulant as it plays a vital role in activating several coagulation factors such as Factor IX, Factor X, Prothrombin, and Factor VII. It is commonly used to reverse the effects of Warfarin-induced hemorrhage in medical settings.^127^ Phosphatidylcholine has also been found to activate the coagulation cascade and accelerate platelet aggregation.^126^ Studies have found that doping vitamin K or electrospinning phosphatidylcholine to a silk fibroin coated BNC/chitosan dressing decreased blood loss and increased blood uptake when compared with a silk fibroin coated BNC/chitosan with no hemostatic agents in a diabetic bleeding rat model.^14^

While various minerals, vitamins, and other naturally occurring hemostatic agents have been found to have potential for use in BNC-based hemostatic dressings, a limitation of these studies is the lack of research on the stability of these fabricated dressings. This is an important factor to consider, especially as the majority of the fabrication methods used to produce these hemostatic dressings utilized physical bonding. Therefore, it is suggested that future research should incorporate stability tests in their studies. Additionally, there has been very little research performed within this area of vitamins and minerals as hemostatic agents; therefore, there is great potential in this area of research.

### 3.4 Coating with Synthetic Polymers, Inorganic Compounds, and Therapeutic Drugs

In addition to natural polymers and hemostatic agents, several studies have applied synthetic coatings and inorganic particles to enhance the hemostatic properties of BNC. Zhang et al. studied the use of a multiporous BNC modified with chlorinated *N*-isopropylacrylamide (NIPAM-Cl) to enhance its antibacterial properties (Figure 3D).^58^ NIPAM is a temperature sensitive polymer and therefore has been studied for thermal responsive applications. Additionally, chlorine is a well-known disinfectant; however, the exact mechanism is poorly understood.^128^ To fabricate the membranes, NIPAM was chemically grafted to BNC using triethoxyvinylsilane, followed by immersion in a hypochlorite solution to chlorinate the membrane. The chlorine was rapidly released from the BNC, with half of the Cl^+^ released within the first 4 h. This quick release is critical to preventing infection. A suspension bacteria assay showed antibacterial efficiency against *S. aureus* and *E. coli*. Additionally, it was found that the addition of the NIPAM-Cl to the BNC significantly decreased the BCI.^58^

Another approach that has been studied to enhance the antibacterial properties of BNC membranes is through the incorporation of a nanocatalyst with glucose oxidase, which can decompose glucose from blood absorbed in the BNC membrane, generating hydroxide radicals for bacteria eradication. The addition of the nanocatalyst showed high antibacterial efficiency against *S. aureus* and *E. coli* in a bacterial suspension assay as well as a crystal violet biofilm assay when compared with a BNC membrane without the nanocatalyst. Additionally, the BNC membranes with the nanocatalyst exhibited low BCI, reduced platelet and RBC adhesion, and minimized blood loss in a bleeding rat tail model when compared to BNC without the nanocatalyst.^129^

For the dual action of promoting hemostasis, followed by promoting healing of the wound through angiogenesis, BNC was grafted with desferrioxamine (DFO), an angiogenic drug. DFO was chemically grafted on an OBNC sponge through amine bonds and was found to promote clot formation and had a significantly lower blood clotting time and lower blood loss when compared with a clinically used collagen sponge. It also enhanced proliferation of HUVECs and the secretion of hypoxia-inducible factor 1-alpha, promoting vascularization.^110^

While several studies have been performed to investigate various modifications of BNC to enhance its hemostatic potential, there are several limitations of these studies, and more thorough studies of different modifications and methods of modification is required. Several of the modifications discussed in this section include physical grafting rather than chemical grafting, where issues can arise due to grafting stability. Another limitation of these studies is that the loss of bioactivity over time is rarely investigated and could have implications on the effectiveness of the dressing. Overall, the use of BNC for hemostatic applications is an area of research that has large knowledge gaps; however, it has great potential to make advancements to the medical field.

## 4 Bacterial Nanocellulose as Artificial Vascular Grafts

Fundamental research has revealed that BNC-based vascular grafts possess unique properties that make them attractive alternatives to existing synthetic grafts. BNC-based vascular grafts have been shown to exhibit outstanding biocompatibility,^37^ high-burst pressure, nanofibril-like structures,^28^ thermal stability,^47^ and desired surgical manageability.^29,32,130^ Moreover, BNC-based vascular grafts have been found to integrate well with the surrounding tissue and host arteries,^30^ promoting vascular remodeling by endothelial cells and SMCs.^131^ The high-water-retention capabilities of BNC and the hydration layer formed around it result in increased hemocompatibility and limit the activation of the blood coagulation system.^37,47^ Thrombin generation has been found to be significantly less on BNC vascular grafts than commercially used PET and ePTFE grafts.^34^ These unique properties have led to the fabrication of BNC-based vascular grafts with various dimensions (below 5 mm).^30^

Several modification techniques have been researched to enhance the hemocompatibility and mechanical properties of BNC-based constructs for use in vascular grafts. Moreover, owing to the shapeability of BNC, researchers have explored various fabrication methods to achieve BNC vascular grafts with properties more closely resembling those of native blood vessels.

### 4.1 Enhancing Hemocompatibility through Endothelialization

One of the most researched methods for improving the hemocompatibility of BNC-based vascular grafts is through the endothelialization of the graft material. As a result, significant research efforts have been devoted to developing various modifications to BNC that increase cell attachment and proliferation.^101^ Endothelial cells express innate anticoagulant properties; therefore, the presence of a confluent endothelial layer could enhance the hemocompatibility of the surface. This can be achieved through either preseeding the biomaterial with cells *in vitro* or modifying the surface with appropriate biomarkers and bioactive agents to recruit native endothelial cells *in vivo*. There are three proposed mechanisms in which *in vivo* endothelization occurs: (1) human endothelial progenitor cells (EPCs) attach, proliferate, and differentiate on the graft, (2) endothelial cells migrate from the native vessel onto the graft, and (3) endothelial cells migrate from surrounding capillaries onto porous vascular grafts.^32,132,133^ Mature endothelial cells typically have low proliferation potential; therefore, the recruitment of EPCs from peripheral blood is thought to produce better endothelization of vascular grafts.^32,133,134^ Several modifications have been researched to enhance cell attachment, migration, and proliferation in BNC vascular grafts, through functionalization with anticoagulant agents,^32,43,135−137^ adhesion peptides,^41,138−140^ and proteins,^32,105,141^ through inducing magnetic forces using metal nanoparticles,^142−144^ and through altering the BNC microstructure.^27,145^

#### 4.1.1 Heparin Immobilization

Extensive research has been conducted on creating blood-contacting biointerfaces with antithrombotic properties by attaching anticoagulants either alone or in conjunction with other bioactive and bioinert molecules. Heparin is a glycosaminoglycan and a well-known anticoagulant that inhibits the coagulation cascade by binding with antithrombin, enhancing its enzymatic activity, which in turn inhibits thrombin, preventing thrombosis.^146^ Heparin also binds with several other proteins, such as the fibroblast growth factor, endothelial cell growth factor, and vascular endothelial growth factor (VEGF). This binding is hypothesized to prevent these growth factors from acid or heat inactivation, as well as increasing the affinity of growth factors to its cell surface receptors and promoting secretion of growth factors from native cells.^147−151^ Heparin has also been found to inhibit the growth of fibroblasts and to modulate the attachment of SMCs, which is critical in preventing intimal hyperplasia.^32,137,152^ The body naturally prevents the overgrowth of SMCs on the vascular wall, as the native endothelium produces heparin-like substances that modulate smooth muscle cell growth.^151^ In addition to its innate antithrombotic properties, it has been found that heparin also promotes the growth of human umbilical vein endothelial cells (HUVECs).^151^ These findings were supported in a study by Bao et al. which found that BNC/silk fibroin tubes chemically functionalized with heparin using 1-ethyl-3-(3-dimethylaminopropyl)carbodiimide/*N*-hydroxysuccinimide (EDC/NHS) cross-linking not only had greater proliferation of HUVECs but also had a lower proliferation of human SMCs when compared to control BNC and BNC/silk fibroin tubes. Hemocompatibility tests performed before endothelization showed reduced platelet adhesion compared to BNC/silk fibroin tubes without heparin as well as compared to PTFE.^136^ Similarly, another study found significantly lower platelet adhesion on heparin-modified electrospun BNC/cellulose acetate (CA) grafts compared to unmodified BNC/CA grafts as well as CA grafts.^43^ Endothelialization was also shown to be enhanced in BNC modified with heparin when compared to unmodified control BNC samples in a study using pig iliac endothelial cells (PIECs),^135^ as well as EPCs.^32^ Thrombogenicity has also shown to be significantly reduced in a whole blood clotting assay with the addition of heparin to a BNC tube (measured before endothelization).^135^ Despite the promising results obtained from these studies, there are several limitations associated with heparin coated surfaces. As mentioned above, heparin binds not only to antithrombin but also to other plasma proteins, resulting in nonspecific interactions that can decrease the efficiency and bioactivity of the coating. In addition, the heparin coating is susceptible to depletion and leaching over time, which may lead to a gradual loss of the coating’s anticoagulant and cell-binding properties.^153,154^ Another limitation of these studies is that the investigation of hemocompatibility was conducted before endothelization of the grafts. As a result, the impact of hemocompatibility on the endothelialized grafts and the effectiveness of the endothelial layer in preventing clot formation remain unclear.

#### 4.1.2 Adhesion Peptides

To enhance cell attachment and proliferation on BNC membranes, researchers have investigated the use of adhesion peptides that contain amino acid sequences present on ECM proteins. These sequences have been found to facilitate cell attachment, migration, and proliferation through binding to integrin receptors on the cell surface.^41^ The most common sequence used is arginine-glycine-aspartic acid, also known as RGD, due to its affinity to multiple cell adhesion receptors and its potent biological activity.^139^ Variations of this sequence, such as glycine-arginine-glycine-aspartic acid-serine (GRGDS) and glycine-arginine-glycine-aspartic acid-tyrosine, have also been investigated.^39,41^ These peptides can then be linked to BNC through functionalization with a cellulose binding module (CBM). CBMs are protein modules that form the domains of cellulose degrading enzymes, therefore providing a high affinity for binding to cellulose and a low affinity for nonspecific adhesion of proteins. In addition, they are biocompatible and inexpensive, making them ideal for medical applications.^155,156^ Studies have shown that the conjugation of RGD with a CBM domain to BNC enhanced cell growth, proliferation, distribution, and elongation in various cell types including fibroblasts, HMECs, neuroblasts, and mesenchymal stem cells. Consistent findings from these studies indicate that cells cultured on untreated BNC exhibited a round morphology and tended to aggregate, suggesting the preference for cell-to-cell attachment over the attachment to the biomaterial, while cells cultured on RGD-CBM functionalized BNC were elongated in morphology.^41,138,139,157^ A study by Weishaupt et al. combined the use of an antimicrobial peptide sequence fused to a CBM as well as RGD-CBM conjugated to BNC and found significantly higher cell spreading of normal human dermal fibroblasts and significantly decreased bacteria concentration when compared to unmodified BNC.^140^

Another method that has been explored to conjugate adhesion peptides to cellulose is fusion with xyloglucan (XG), a polysaccharide found in most vascular plants. XG naturally binds noncovalently to cellulose, which is advantageous over CBMs as it does not alter the BNC structure. BNC membranes functionalized with XG-RGD as well as XG-GRGDS were found to enhance proliferation of human saphenous vein endothelial cells (HSVEC) *in vitro*.^39,134^ While the purpose of these studies was to enhance cell adhesion to BNC for use as vascular grafts, hemocompatibility was not investigated (before or after endothelization).

#### 4.1.3 Extracellular Matrix Proteins

To enhance the biofunctional features of BNC membranes and to produce a functional endothelial layer, different ECM proteins such as fibronectin, albumin, and collagen have been used.^32,105,141^ Wacker et al. studied the effect of coating BNC with either albumin or fibronectin for the adhesion and proliferation of EPCs and HSVECs. The albumin and fibronectin were cross-linked using 1-cyano-4-dimethylamino pyridinium tetrafluoroborate (CDAP) to generate cyanate ester derivatives, followed by bioconjugation. They found that fibronectin significantly increased cell adhesion and proliferation while albumin showed no difference when compared to untreated BNC.^32^ Further, chemically cross-linking gelatin using glutaraldehyde to BNC tubes has shown to significantly increase HUVEC and SMC attachment and proliferation compared to untreated BNC.^105^ The addition of gelatin to the tubes had no significant effect on whole blood clotting time (measured before endothelialization). Additionally, Kuzmenko et al. conjugated fibronectin to BNC for the expansion of HUVECs and conjugated collagen to BNC for the expansion of MSCs, using CDAP for both bioconjugations. Both HUVECs and MSCs were found to have significantly higher cell proliferation and cell elongation when grown on conjugated BNC than untreated BNC.^141^ No tests were performed to assess the effect of the conjugation of these proteins on the hemocompatibility of the BNC. There are several limitations when using ECM proteins for enhancing endothelialization of BNC vascular grafts. While ECM proteins are less likely to promote a foreign body response than synthetic materials, if these proteins are sourced from allogenic or xenogeneic donors, there is a potential to cause an immune response.^158^ Additionally, these proteins are not cell specific and have the potential to promote adhesion of other cell types; therefore, these coatings are more appropriate when the grafts are to be preseeding with cells followed by implantation.

#### 4.1.4 Structural Modifications

**Figure 4:**
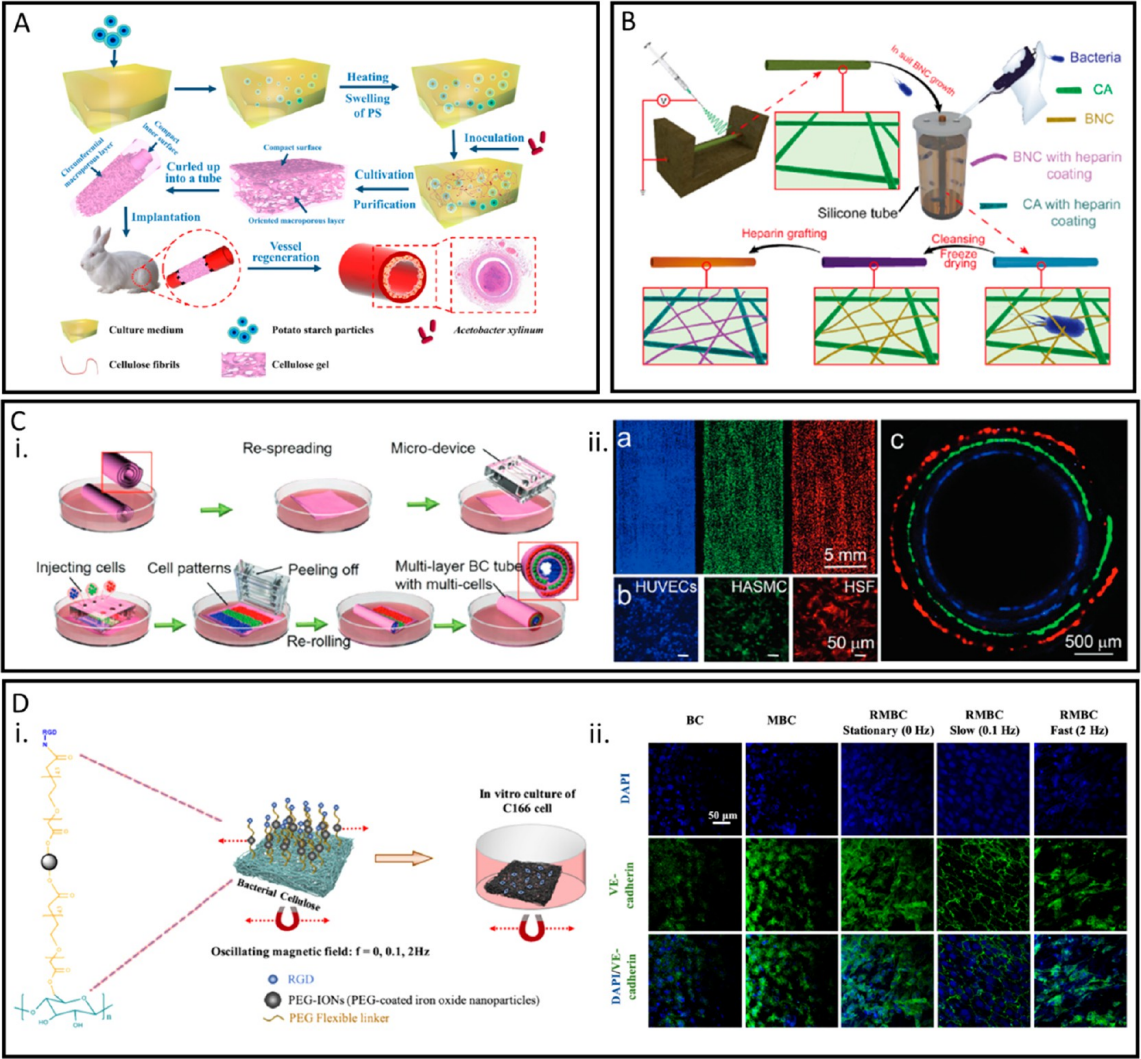
Different modification techniques to enhance the cytocompatibility and cell-adhesion properties of BNC. (A) Potato starch particles were added *in situ* during BNC synthesis to produce greater porosity within the BNC to enhance cell migration and growth within the construct. Figure reprinted with permission from ref (27). Copyright 2022 Elsevier. (B) BNC was produced within cellulose acetate fibers to add a layer of submicron fibers to increase endothelization. Figure reprinted with permission from (43). Copyright 2021 Elsevier. (C) (i) Shape memory tubular BNC was produced to allow for unrolling the tube for patterned cell adhesion, followed by rerolling the tube for implantation. (ii) Shape memory tubular BNC stained for HUVECs, HASMCs, and HSFs, present in different layers of the BNC tube. (a) and (b) are before rolling, and (c) is after rolling. Figure reprinted with permission from ref (161). Copyright 2017 John Wiley and Sons. (D) (i) A magnetic field was produced on BNC using iron oxide nanoparticles to enhance cell adhesion. (ii) Endothelial cells (C166 line) stained with vascular endothelial cadherin (green) and nuclei (blue) cultured on BNC, magnetic BNC (MBC), and RGD-grafted magnetic BNC (RMBC). The RMBC was subjected to oscillation frequencies of 0, 0.1, and 2 Hz. Figure reprinted with permission from ref (142). Copyright 2020 American Chemical Society.

BNC is a highly porous hydrogel. The pore size and fiber diameters of BNC membranes can differ depending on culture conditions, bacteria source, and postmodification and purification procedures. Unmodified BNC membranes have shown to prevent the infiltration of cells due to their relatively small pore sizes (<1 μm) in their nanofibrous structure.^159^ However, several modification techniques have been employed to tune the 3D structure of BNC and increase its pore size to allow for a higher integration of cells. One method to generate 3D BNC is through the addition of insoluble beads into the culture media during BNC synthesis, followed by removal through melting or addition of a surfactant.^27,145^ In a study comparing paraffin beads with diameters of 290, 136, and 61 μm, 61 μm beads were found to promote a higher concentration of fibroblast cell infiltration when compared with other bead sizes and unmodified BNC. The bead diameters of 290 and 136 μm generated very large pore sizes of >150 μm where the 61 μm diameter bead generated pores <80 μm. This study did not analyze the mechanical properties of the 3D BNC; however, the generation of large pores has the potential to affect the mechanical integrity of the constructs, which was observed in a study which added swollen potato starch beads of approximately 50 μm diameter to BNC culture to create pores of 20–40 μm (Figure 4A). It was found that the pores decreased the Young’s modulus of the material. However, the addition of the pores enhanced the HUVEC and fibroblast cell infiltration. The hemocompatibility of the 3D BNC was tested, and while platelets were found to adhere to the surface, they were not in an activated state.^27^

The innermost layer of blood vessels is termed the tunica intima, which consists of an endothelial layer attached to a basement membrane. The native basement membrane consists of a network of 3D woven fibers on the nano- (1–100 nm) and submicrometer (100–1000 nm) scale. BNC fibers are typically 20–100 nm in size, fitting the criteria for the nanoscale; however, they lack the submicron fiber scale. Therefore, studies have incorporated CA fibers with BNC to add the submicron fiber layer (Figure 4B). Initially, CA was electrospun onto a cylinder receiver to form a graft. A silicone tube bioreactor was then used to synthesize BNC inside the CA graft through the addition of bacteria and culture media. The resulting BNC/CA graft was found to have increased endothelialization when seeded with HUVECs as well as a greater tensile strength and Young’s modulus when compared to the CA as well as the BNC graft. Platelet adhesion (measured before endothelization) was found to be significantly higher in the CA/BNC graft compared to the CA graft; however, it was lower than that in the BNC graft. Additionally, biocompatibility of the CA/BNC graft was verified with both *in vitro* and *in vivo* biocompatibility assays.^43,160^

Another modification that has been performed on the BNC to enhance its cell adhesion properties is the process of mercerization. This process involves the addition of an alkaline solution that transforms cellulose I to cellulose II. A study by Hu et al. used concentrations of 10%, 15%, and 20% w/v solutions of NaOH to soak BNC tubes and found that the fiber diameter and porosity increased with increasing concentrations of NaOH, yielding a higher infiltration and proliferation of HUVECs. No significant differences were found in blood clotting assay or plasma recalcification when comparing mercerized to unmodified BNC, measured before endothelization. Finally, the *in vivo* biocompatibility in a rat model indicated that the mercerized BNC tubes have considerable potential for use as artificial blood vessels.^102^

To overcome the additional complexity of cell seeding on modified BNC while in tubular form, a study by Li et al. induced stress to form shape memory in BNC membranes to allow them to self-roll into tubular structures (Figure 4C). To produce this shape memory BNC membrane, a BNC pellicle was rolled around a mandrel, lyophilized, and then removed from the mandrel. The rolled BNC membrane was then unrolled, and HUVEC, human aortic smooth muscle cells (HASMC), and fibroblasts were seeded in patterns using microfluidic chips. The BNC membrane was then able to self-roll back into a tube, with three different layers of cells, representative of the different cell layers present in a blood vessel; however, no hemocompatibility testing was performed on these grafts.^161^

#### 4.1.5 Magnetic Forces

To enhance attraction between cells and BNC, magnetic forces have been utilized, as they have been shown to promote adhesion of various cell types.^142,143^ In this approach, the BNC membrane is magnetized through the addition of iron oxide nanoparticles (IONs). The target cells can also be magnetized by either electroporation or passive uptake of IONs to enhance the attractive forces. The IONs can be either uncoated or coated in PEG or dextran to increase biocompatibility.^142,144,162^ A study by Zhang et al. synthesized a magnetic BNC membrane with RGD peptides to have the dual action of attracting cells through magnetic forces and aiding attachment using an adhesion peptide (Figure 4D). Carboxylated PEG was functionalized with IONs to form PEG-IONs through thermal decomposition. The PEG-IONs were used to coat BNC using Steglich esterification to endow the BNC with magnetic properties. Furthermore, EDC/NHS chemistry was used to conjugate RGD peptides to the surface of the magnetized BNC. This resulted in enhanced adhesion and proliferation of murine endothelial cells when compared to untreated BNC and magnetized BNC without RGD.^142^ Another method of magnetizing BNC involved the addition of Fe^3+^ and Fe^2+^ iron salts into culture media, which form IONs during BNC synthesis. HASMCs were magnetized with dextran coated IONs through passive uptake, and a parallel plate flow chamber with a magnetic field was used to seed the magnetized BNC. However, the magnetized BNC was not found to significantly improve cell attachment or proliferation when compared to unmodified BNC.^128^ While these studies tested the effect of magnetic forces on the cell adhesion and proliferation to BNC membranes, no hemocompatibility tests were performed. Studies have shown that magnetic fields can affect blood flow through orientation of blood cells and can potentially limit thrombosis through promoting blood flow.^163^ Additionally, studies have shown that magnetic nanoparticles reduce platelet aggregation.^164^ Therefore, an important extension of this research would be testing magnetized BNC for thrombogenicity.

### 4.2 Enhancing Mechanical Properties through Chemical Modification and BNC Tubular Fabrication

In addition to excellent hemocompatibility and cell adhesion properties, an important design consideration when fabricating an artificial vascular graft is to match the mechanical properties between the graft and the native blood vessel. A native vein has been found to have an average longitudinal Young’s modulus of around 25 MPa, a longitudinal stress at break of around 6 MPa, a longitudinal strain at break of around 83%, and a bursting pressure of around 1700–4000 mmHg. In contrast, ePTFE, a commonly used graft material, has been found to have a longitudinal Young’s modulus of 500 MPa, a longitudinal stress at break of 14 MPa, and a bursting pressure of 600 mmHg, which is significantly lower than that of a native vein; however, typical pressures within veins do not exceed 100 mmHg.^165^ Compliance mismatch is a common and serious issue associated with artificial vascular grafts. This can lead to intimal hyperplasia, which occurs when there is a change in wall shear stress between the graft and native tissue, causing the body to initiate thickening of the vessel wall.^23^ To minimize these complications, it is essential to match the compliance of the graft as closely as possible to that of the native vessel tissue. The compliance of a typical vessel is around 2%, whereas ePTFE has a compliance of about 0.2%.^133^ Extensive research has been conducted investigating the use of BNC as vascular grafts due to it possessing mechanical properties similar to those of blood vessels. Several studies have found that unmodified BNC has similar, however slightly lower, mechanical properties compared to native vascular tissue;^102,166^ therefore, studies have further aimed to enhance and alter the mechanical properties of BNC constructs through different modification and fabrication techniques. Additionally, the shapeability of BNC during biosynthesis allows tubes of different diameters and lengths to be produced, depending on the required application. Other common limitations of engineered vascular grafts are the ability for the graft to have the appropriate bursting strength as well as elasticity.^162^ These limitations have been addressed by various studies altering the mechanical properties of the BNC through various fabrication methods or chemical modifications.

There are several different methods that have been used to fabricate tubular BNC, each with different advantages and disadvantages, yielding BNC tubes with varying properties (Table 1). The pellicle formation method involves producing tubular BNC initially as a pellicle before shaping it into a tube. Other methods of tubular fabrication use tube shaped fermenters consisting of tubes of glass or oxygen permeable materials arranged in either a vertical or horizontal orientation. If no gas exchange occurs, then it is considered static fermentation. If there is continuous gas exchange within the fermenter, then dynamic fermentation. A less used method of tubular fabrication involves cyclical fermentation methods.

**Table 1:** Properties of Tubular BNC Fabricated Using Different Methods

		Tubular Fabrication		
	Pellicle Fermentation	Static Fermentation	Dynamic Fermentation	Cyclical Fermentation
Culture Method	Static culture of pellicles, then formed into a tube	Culture around rods (inner or outer side) without any gas flow	Culture around oxygen permeable rods with a steady state flow of oxygen	Fermentation around an inert tube cyclically dipped in medium
Dimensions				
Thickness (mm)	2–2.4^5,161^	0.4–1.2^8,36,170,179^	0.2–2.6^28,66,102,138,160,166,180−183^	0.5–3^131,167,174^
Inner Diameter (mm)	0.5–10^5,161^	1–8^8,36,179^	3–12^28,66,138,160,181,184^	1–3^131,174^
Mechanical Properties				
Axial Tensile Strength (MPa)	2.75^5^	0.6–0.8^36,135,170,179^	0.35–2^28,66,102,138,160,166,180,181,183,184^	
Young’s modulus (MPa)	10.5^5^	0.06–3.2^36,135,179^	0.25–6^43,47,66,102,138,160,166,181^	
Elongation at Break (%)		50–65^135^	20–50^43,66,138,160,166,181^	
Suture Retention (N)	3.9–4.2^5^	0.5^37^	0.15–0.7^66,102,166,184^	4–8^6,30^
Burst Pressure (MPa)		0.03–0.11^37,135^	0.01–0.25^37,66,102,166^	0.09–0.11^30,167^
Compliance (%/mmHg)	0.04^5^		0.043^28^	
Advantages	Simple method	Uniform in structure	Uniform in structure	Uniform in structure
	Easier cell culturing	Able to be equipped with reinforcing structures	Reproducible and highly efficient process	Anisotropic orientation of BNC fibers
			Able to be equipped with reinforcing structures	Reproducible and highly efficient process
Limitations	Nonuniform in structure	Dependent on oxygen pressure	Dependent on oxygen flow	Complicated setup
		Restriction in achieving large thicknesses		
		Time consuming and low efficiency process		

#### 4.2.1 Producing Tubular BNC from Pellicles

**Figure 5:**
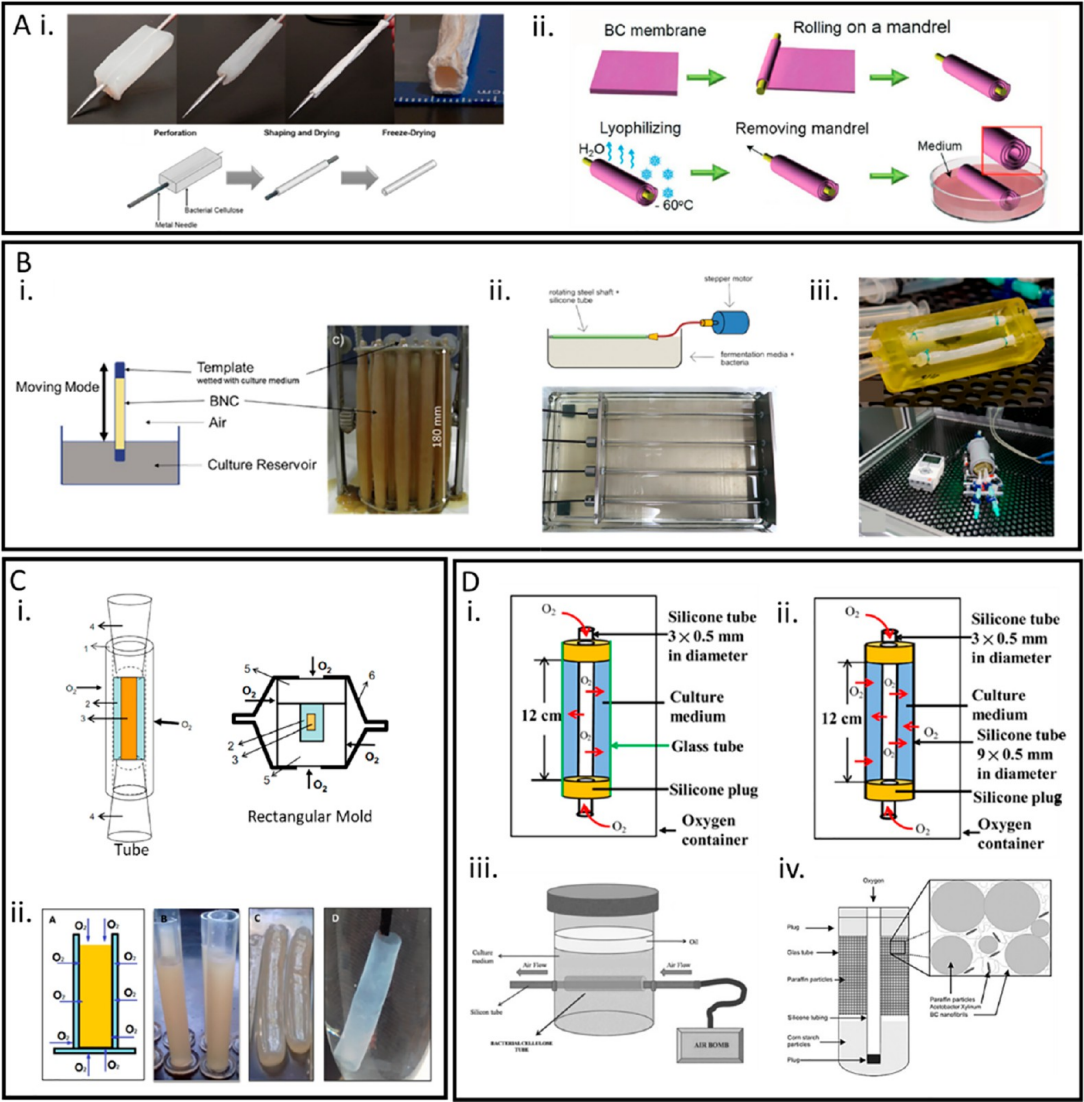
Different fabrication methods used to produce tubular BNC. (A) Pellicle fermentation methods. (i) Fabrication of tubular BNC using a BNC block, which is perforated and dried around a needle. Figure reprinted with permission from ref (5). Copyright 2016 John Wiley & Sons. (ii) A schematic of the fabrication of a tubular BNC membrane using a BNC pellicle rolled around a mandrel, which is then lyophilized and the mandrel then removed. The rolled tube can be unrolled for seeding of cells; however, it possesses shape memory to recover tubular shape for use as an artificial vascular graft. Figure reprinted with permission from ref (161). Copyright 2017 John Wiley and Sons. (B) Cyclical fermentation methods. (i) Glass or bamboo templates are moved in and out of culture media at set intervals to produce tubular BNC. Figure reprinted with permission from ref (167). Copyright 2021 Elsevier. (ii) Silicone tubes covering steel shafts attached to a steeper motor are placed on the surface of culture media and rotated at 30 rpm to produce tubular BNC. Figure reprinted under a Creative Commons Attribution License (CC BY) from ref (131). Copyright 2022 Fusco, Meissner, Podesser, Marsano, Grapow, Eckstein and Winkler. (C) Static fermentation methods. (i) Schematic of a tube shaped bioreactor and a rectangular mold used to fabricate tubular BNC: (1) wall of the silicone tube, (2) bacterial cellulose, (3) culture media, (4) plug, (5) rectangular silicone mold, (6) clamp. Figure reprinted with permission from ref (36). Copyright 2008 Elsevier. (ii) Vertical PDMS molds are placed within a chamber of culture media with no air/oxygen added. Figure reprinted with permission from ref (170). Copyright 2021 Elsevier. (D) Dynamic fermentation methods. (i) Schematic of a bioreactor consisting of an inner silicone tube with oxygen flowing through it, contained within a glass tube. (ii) Schematic of a bioreactor consisting of a silicone inner tube with oxygen flowing through and an outer silicone tube in which oxygen can permeate into. Figure reprinted with permission from ref (37). Copyright 2020 Elsevier. (iii) Schematic of a horizontal silicone tube with air flowing through inside a chamber of culture media. Figure reprinted from ref (138). Copyright 2013 Fábia K Andrade et al. (iv) Schematic of a bioreactor with an inner silicone tube with 100% oxygen flowed through within a glass tube. Starch and paraffin particles were added to the culture media to increase porosity in the BNC tube. Figure reprinted with permission from ref (9). Copyright 2008 John Wiley and Sons.

A straightforward method to create a tubular BNC involves fabricating it as a flat pellicle, followed by shaping it into a tubular form (Figure 5A). This method offers several advantages such as simplified experimental setup and easier access to the lumen surface for cell culture and blood interaction studies.^27,161^ However, the tubes produced using this method are typically nonuniform and exhibit lower shape holding ability as they were initially fabricated as a sheet. Leitão et al. proposed perforating a needle through a BNC block and then shaping and drying the block around the needle to form a tube-shaped BNC. The grafts were implanted in a pig model, and after 1 month, patency was achieved, and neo-natal and endothelial cell attachment was observed on the inner surface of the BNC membrane.^5^ Another method for pellicle fermentation to produce tubular BNC involves the formation of a multilayered BNC pellicle, which is then rolled onto a mandrel and lyophilized before removal from the mandrel.^161^ This method creates a multilayered membrane that could be ideal for mimicking the multilayered structure of native vascular tissues.

#### 4.2.2 Cyclical Fermentation Methods of Producing Tubular BNC

Few studies have utilized cyclical fermentation methods (Figure 5B) in which tubes are moved in and out of the culture media at set intervals. Klemm et al. developed a method known as Mobile Matrix Reservoir Technology to address the issue of gradient formation during the BNC pellicle fabrication, which occurs due to a denser zone at the air interface and a less dense zone at the culture media interface. This technology has also been translated to tubular BNC fabrication.^167,168^ This method involves moving bamboo or glass templates wetted with culture media into and out of a culture reservoir. The tubes are then manually inverted so that the smooth outer layer becomes the inner layer.^169^ Tubes of wall thicknesses up to 3.3 mm with burst pressures of up to 750 mmHg were produced using this method. However, when these BNC tubes were tested for hemocompatibility by flowing blood through the tubes, there were no significant differences when compared to PET or ePTFE in terms of thrombocyte adhesion and activation.^169^

Another cyclical setup used cylindrical bamboo templates that were rotated within a PVC pipe with media flowing through; however, the mechanical properties of these tubes were not investigated. A similar setup had also been used with silicone tubes around a steel shaft attached to a stepper motor within a vessel of culture media. The stepper motor was set at 30 rpm to allow for continuous rotation of the tube on top of the culture media. The synthesized tubes were tested in a large animal model, and after 4 weeks, the tubes were completely endothelialized *in vivo* and compete patency was observed.^131^

#### 4.2.3 Static Fermentation Methods of Producing Tubular BNC

Static fermentation methods for fabricating tubular BNC involve the use of tube-shaped oxygen permeable fermenters, typically consisting of one or two concentric tubes of glass or oxygen permeable materials such as polydimethylsiloxane (PDMS) (Figure 5C).^170^ The first known static fermentation method developed for BNC tubes used a glass tube surrounded with culture media within a glass chamber to produce tubular BNC, with several variations of this setup having been patented.^6−8,30,168,171−174^ The resulting BNC tubes were termed BActerial SYnthesized Cellulose (BASYC). While these tubes were generally uniform, the process is time-consuming and is unable to produce tubes longer than 20 mm.^6,7,175^ In animal studies, BASYC grafts were implanted in the jugular veins of 10 rats. The analysis showed high stability with normal blood flow and the formation of connective tissue around the implants as well as no stenosis and thrombosis after three months.^174^ In another study, primary results from implanted BASYC grafts in carotid arteries of eight pigs showed rapid endothelialization and regeneration of vascular tissue with no need for precell seeding; however, their low patency remained a challenge.^7^

To overcome the limitation of the short length of the BASYC tubes, Bertholdt et al. patented a device to produce long tubes in which a glass rod was placed within two half pipes on the surface of an early stage BNC pellicle, allowing for the BNC to grow into the gaps, forming a tube-shape membrane.^173^ However, no studies were found that utilized this device to produce tubular BNC.

A common static fermentation method for producing tubular BNC involves the use of plugged silicone molds with culture media inside, orientated either vertically or horizontally. The use of a silicone mold rather than glass allows for oxygen to permeate through.^47,170^ The inner diameter of the molds determines the outer diameter of the BNC tubes. A study by Putra et al. compared different diameters of silicone tubes using this setup as well as the use of a rectangular silicone mold instead of a tube. This study found that fibrils were orientated in silicone tubes with a diameter of <8 mm, compared to no fibril orientation occurring within the rectangular mold. They also found that oxygen availability affected the rate of production of the tubular BNC.^36^ Other studies have shown this fermentation method produces tubes with desirable hemocompatibility, as synthesized tubes had a blood clotting time greater than 60 min and a hemolysis rate less than 0.5%. However, the resulting tubes had poor mechanical properties, with a much lower Young’s modulus and stress at break than native veins.^47^ A study by Piasecka-Zelga et al. used a tubular PDMS mold, orientated horizontally with culture media inside to produce BNC tubes with chitosan, by adding chitosan oligomers to the culture media during BNC synthesis. The synthesized BNC/chitosan vascular grafts were analyzed on 30 rats and 15 guinea pigs in terms of potential toxicity and long-term stability to replace small blood vessels without thrombogenicity. The result indicates no signs of toxicity, immune reaction, or hyperplasia of the muscle tissue surrounding the implant in the animals.^103^ A modification of this experimental setup used a glass rod within a silicone tube, with the BNC synthesized between the two tubes, allowing oxygen to permeate from the outside into the silicon tube. The synthesized tubes had a very smooth inner surface, resulting in very low platelet adhesion to the BNC tube.^37^ Additionally, the effect of oxygenating the culture media before BNC synthesis was investigated and it was found that denser tubes with a higher concentration of bacteria closer to the silicone support were produced when the culture media was oxygenated compared to nonoxygenated culture media.^104^

Due to oxygen limitations and gradients occurring in static fermentation methods of tubular BNC production, generally this method produces tubes with a multilayered structure with poor mechanical properties. This method is also time-consuming and limited in the thickness and length of tubes that can be produced.^166^

#### 4.2.4 Dynamic Fermentation Methods of Producing Tubular BNC

Dynamic fermentation methods have shown to be more effective than static fermentation in producing tubular BNC due to an increase in oxygen delivery during bacterial synthesis of BNC.^166^ Several different dynamic fermentation methods have been developed for producing BNC vascular grafts (Figure 5D). One method of dynamic fermentation is a horizontal setup that employs an inner silicone tube submerged in a glass tube with air or oxygen flowing through. Bodin et al. studied the effect of adding different concentrations of oxygen (21%, 35%, 50%, and 100%) within the inner silicone tube and found that the thickness of the tube increased with an increase in oxygen concentration. A tube thickness of 0.51 mm was achieved using 20% oxygen compared with 1.30 mm using 100% oxygen. Additionally, a higher oxygen concentration resulted in denser tubes, with a less noticeable layered structure, and a higher burst pressure, with the 100% oxygen conditions have tubes with a burst pressure over 800 mmHg, compared to only 250 mmHg when 20% oxygen was used.^176^

To allow for greater oxygen delivery to the bacteria during BNC syntheses, a vertical bioreactor with an inner silicone tube with oxygen flowing through and an outer silicone tube within an oxygen chamber was investigated. Dissolved oxygen concentrations were found to be 4× higher after two weeks of BNC synthesis when oxygen was supplied in the culture media from both directions.^35^ This resulted in dense BNC tubes with a nonlayered structure and enhanced mechanical properties being produced.^35,105,166,175^ A study by Bao et al. compared three different types of vertical tube bioreactors each with varying oxygen flow directions. The single silicone tube bioreactor (S-BNC) had an inner silicone tube within a glass tube, and oxygen permeated through the inner silicone tube into the culture media. The double silicone tube bioreactor (D-BNC) had an inner silicone tube within an outer silicone tube, and the oxygen permeated both the outside and the inside. These bioreactors were compared to a static fermentation method with an inner glass rod (G-BNC) within a silicone tube, and the oxygen permeated through the outer silicone tube into the culture media. The BNC tubes produced in these three different bioreactors exhibited vastly different mechanical properties, with the D-BNC tube having the greatest axial ultimate tensile stress, burst pressure, and suture retention. Meanwhile, the G-BNC tube had the smoothest lumen surface and axial ultimate tensile stress comparable to those of the D-BNC tube. The tubes produced in the S-BNC bioreactor had significantly poorer mechanical properties.^37^ Other studies by Tang et al. and Hong et al. verified these results.^135,166,175^ It was also found that using 100 g/L fructose in the D-BNC bioreactor greatly increased the bursting pressure compared to that when 50 g/L glucose was used in the media. However, the change of sugar source and concentration did not affect the bursting pressure of the tubes produced in the S-BNC bioreactor.^35^ The G-BNC tubes had the greatest hemocompatibility, with the least platelet adhesion, likely due to the smooth lumen surface. The D-BNC tubes had significantly less platelets adhered and a smoother lumen surface than the S-BNC bioreactor.^37^ Moreover, BNC tubes produced in a G-BNC bioreactor were air-dried and compared to wet BNC tubes. The air-dried BNC had a much higher Young’s modulus, tensile strength, and suture retention.^66^

Another study incorporated the use of starch and paraffin particles to increase the porosity within the BNC tube and allow for cell migration into the tube. A vertical bioreactor was used with an outer glass tube and an inner silicone support with 100% oxygen supplied throughout the culture. After the tubes were synthesized, the particles were leached out. The starch particles were smaller than the paraffin particles, and due to differences in density between the two particle types, differently sized pores were created in various areas of the tube. The addition of the particles did not affect the maximum stress at break of the synthesized tubes; however, the Young’s modulus was significantly decreased from 8.25 to 5.97 MPa when compared to unmodified BNC tubes. SMCs were grown on the BNC tube and found to attach to the tube and partially proliferate into the scaffold.^9^

Generally, BNC vascular grafts produced using dynamic fermentation methods had superior mechanical properties compared to grafts produced using static fermentation methods due to the increase in oxygen delivery to the bacteria. Additionally, dynamic fermentation methods allowed for greater tunability of the tube thickness and length. However, the mechanical properties were still slightly lower than native tissue; therefore, there is still a great need for further research within this area.

#### 4.2.5 Chemical Modification

Most studies have focused on the fabrication method to produce BNC tubes with appropriate mechanical properties for vascular grafts; however, a few studies have instead chemically modified or cross-linked the BNC during or after cultivation to enhance its mechanical properties. The addition of cellulose acetate to BNC has shown to increase the tensile strength and Young’s modulus due to hydrogen bonding and mechanical entanglement between the BNC and CA. This resulted in properties more closely matching those of native blood vessels when compared to unmodified BNC membranes.^43,160^ Additionally, the thinner fibers produced with the addition of CA were found to improve the hemocompatibility properties of BNC by decreasing platelet adhesion when compared to larger diameter fibers of unmodified BNC membranes.^160^

A further study introduced poly(vinyl alcohol) (PVA) into the BNC network. The BNC/PVA grafts were found to have a significantly greater tensile strength of 0.45 MPa compared to 0.1 MPa of unmodified BNC. Additionally, the Young’s modulus increased from 0.5 to 3.0 MPa with the addition of PVA to the BNC tube. Burst pressure and suture retention were also significantly increased.^166^ Mercerization has also been investigated to enhance the mechanical properties of BNC. Membranes modified through this process had a significantly higher Young’s modulus and burst pressure when compared to control samples.^102^ The mercerization process was also found to produce a smoother BNC surface, which resulted in less protein absorption and slower plasma clotting.^102^

## 5 Modification Techniques to Enhance Vascularization of Tissue Engineering Constructs

The shapeability, porosity, and moldability of BNC have also been utilized to fabricate vasculature within a tissue engineering construct. Proper vascularization of tissue engineering constructs is critical in the health and integration of the construct into the body as blood vessels transport nutrients and oxygen to the tissues and remove byproducts.^34^

**Figure 6:**
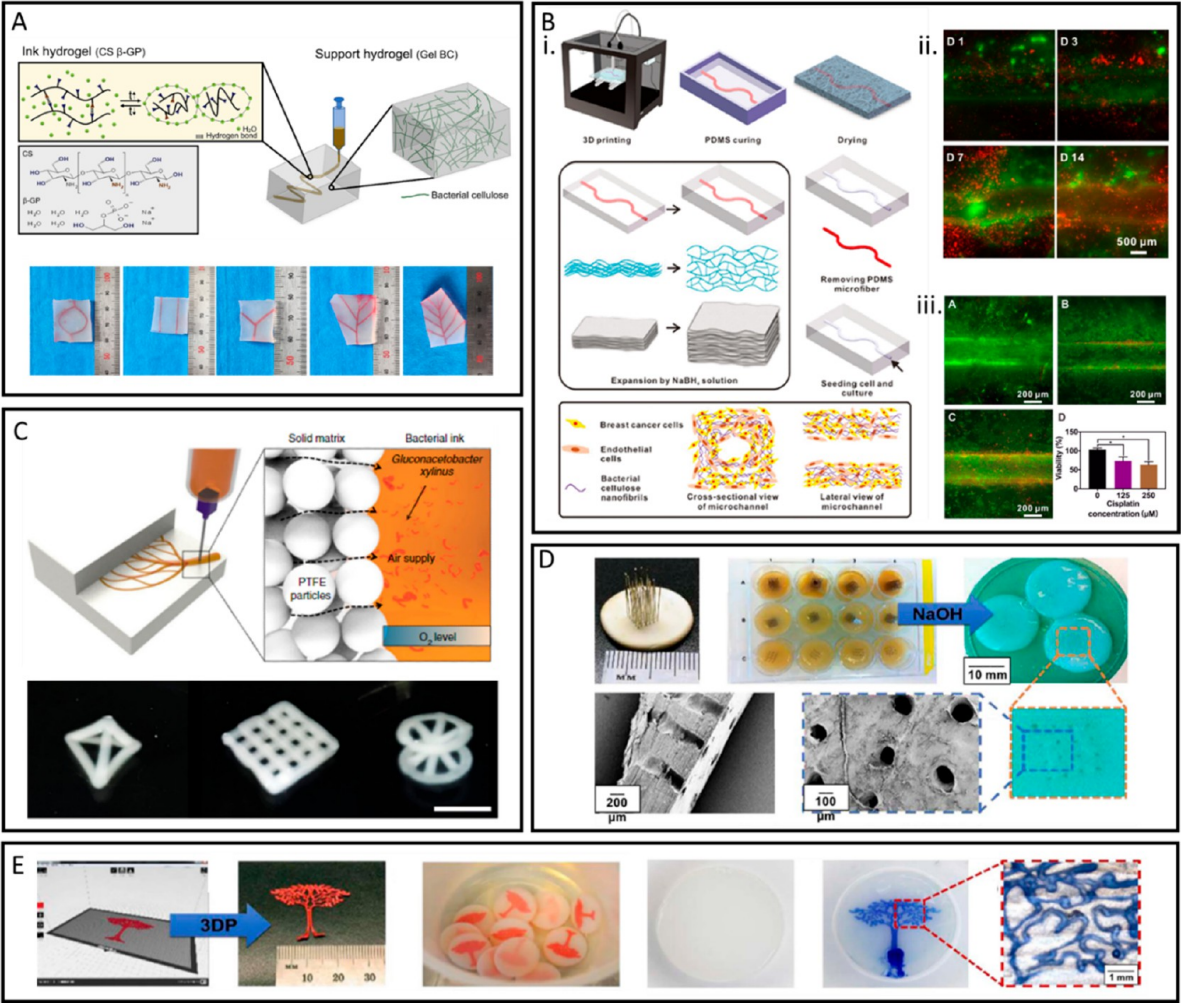
Methods to produce vasculature in BNC for biomimetic tissue engineered constructs. (A) A chitosan/β-sodium glycerophosphate ink hydrogel was used with a gelatin/BNC support hydrogel using 3D printing techniques to produce channels for vascularization. Figure reprinted with permission from ref (10). Copyright 2021 IOP Publishing. (B) To produce a vascularized breast cancer tumor model, PDMS was printed into a BNC scaffold, after which the channel was expanded using NaBH_4_ and the PDMS was removed leaving the channel. The channel was then seeded with MCF-7 and HUVECs. (i) Schematic of the experimental setup. (ii) The channel seeded with MCF-7 (red) and HUVECs (green) at D1, D3, D7, and D14. (iii) The fabricated channel was used as a breast cancer tumor flow model to test drug toxicity (live/dead staining) with (A) 0 μM, (B) 125 μM, and (C) 250 μM cisplatin for 48 h, where (D) shows the cell viability at each concentration of cisplatin. Figure reprinted with permission from ref (11). Copyright 2020 IOP Publishing. (C) A 3D printing method technique was used with ink containing bacteria, incubation media, and cellulose nanofibers and printed into a matrix of PTFE microparticles. The bacteria produced BNC in the printed shape. Image reprinted with permission under a Creative Commons Attribution License (CC BY) from ref (177). Copyright 2019 Sungchul Shin et al. (D) An array of needles was added to culture media with bacteria to create straight channels within the BNC. Figure reproduced with permission from ref (12). Copyright 2019 IOP Publishing. (E) A 3D printed PLA template was added to already forming pellicles to create a network of channels in the BNC. Figure reproduced with permission from ref (12). Copyright 2019 IOP Publishing.

One approach that has been used is through liquid bath 3D printing. In this technique, a gelatin BNC support structure was used with a chitosan/β-sodium glycerophosphate ink. The ink was printed into the gelatin/BNC using a nozzle, and then the gelatin was cross-linked by microbial transglutaminase. The ink was then transformed into a liquid by decreasing the temperature and removed to yield channels of different patterns (Figure 6A). HUVECs were injected and attached and proliferated.^10^ Another 3D printing technique that has been used to produce channels is through 3D printing PDMS into a BNC scaffold, followed by expansion of the channel through gassing with sodium borohydride (NaBH_4_) (Figure 6B). The PDMS is then removed, leaving the channels. They found that the thickness of the channel could be controlled based on NaBH_4_ concentration and expansion time. For this application, HUVECS and MCF-7 cells (a human epithelial cell line isolated from breast cancer) were seeded within the channel to create a breast tumor model with multiscale vascularization.^11^ A 3D printing method in which ink containing bacteria and incubation media (along with cellulose nanofibers) form BNC has also been investigated (Figure 6C). In this technique the ink was printed into a matrix of PTFE microparticles, and following incubation, the bacteria produced BNC in the printed pattern. The advantage of this technique is that free form structures were able to be formed, as methods using gels have limitations based on gels losing structure under gravity.^177^

Other methods of vascularization of BNC involve *in situ* fabrication through the addition of solid structures during BNC synthesis. Sämfors et al. used two such methods to produce vasculature with the first method using an array of vertical standing needles added to media containing bacteria to create straight channels (Figure 6D). The second method produced more complicated network channels by placing 3D printed templates of PLA on top of growing BNC pellicles and allowing the BNC to form around the template. The PLA was then hydrolyzed and removed, leaving a network of channels matching the template (Figure 6E). Both methods were then used to successfully expand HUVECs.^12^

Decellularized matrix in combination with BNC has also been used to create a vascularized tissue construct. In this method, digested and solubilized bladder acellular matrix was combined with BNC nanofibers produced through homogenization followed by TEMPO oxidation, and the resulting matrix was cross-linked and freeze-dried. The construct successfully promoted angiogenesis and epithelization.^178^

While these studies show promising results for the use of BNC as vascularized tissue engineered constructs, there is very limited research in this area, with only a few different techniques being employed. Also, limited testing was performed on these constructs. Future research should focus on further techniques to produce vascularized BNC constructs with thorough testing of the hemocompatibility and biocompatibility of the produced constructs.

## 6 Conclusions

The utilization of BNC as a biomaterial for blood interaction applications holds great promise as a research area, owing to its exceptional properties that enable its fabrication, modification, and functionalization in a diverse array of ways. Functionalized BNC has been found to effectively stop bleeding when used as a hemostatic wound dressing; tubular fabricated BNC constructs have been found to be effective as artificial vascular grafts. BNC has also been explored as a biomimetic tissue engineering construct to fabricate vasculature. Despite the great potential of using BNC as a biomaterial for blood-contacting applications, there is still significant progress that needs to be made before there can be a successful translation from research to clinic. BNC is a natural hydrogel that exhibits tissue-like properties. One important aspect that needs to be considered when using BNC for different blood-contacting applications is to ensure that the surface/bulk modification process does not negatively impact the innate physical and morphological properties of BNC. Postpurification and drying methods used to process BNC can significantly alter its mechanical, structural, and water retention properties; therefore, future studies should take this into consideration and apply the appropriate postprocessing methods to obtain BNC membranes with desired properties. Moreover, when applying this unique natural polymer for vascular tissue applications, we believe future research should focus on developing multifunctional BNC membranes that (1) are hemocompatible, (2) promote targeted binding of cells and angiogenesis, and (3) have desirable mechanical properties that match the patient’s native vascular tissue. Studies have shown in most cases, when targeted cell adhesion is promoted, the hemocompatibility of the biointerface decreases and, when improving the antithrombotic properties, cell adhesion and growth can be limited; therefore, investigating the multifunctional characteristics of the designed membranes is crucial for vascular tissue engineering. Further, hybrid and composite assemblies, which are created by combining two or more materials, possess a unique set of advanced properties that surpass those of their individual components. Therefore, exploring composite BNC materials in combination with effective surface and bulk modification methods could be highly beneficial in obtaining novel BNC constructs with superior properties. Lastly, long-term animal studies and clinical trials need to be conducted to better evaluate the stability of the different coatings, the mechanical strength of the BNC biointerface, and their overall biocompatibility when placed in complex biological environments.

## References

[ref1] World Health Organization: Cardiovascular Diseases. Accessed December 12, 2022. https://www.who.int/health-topics/cardiovascular-diseases.

[ref2] Curry N.; Hopewell S.; Dorée C.; Hyde C.; Brohi K.; Stanworth S. The acute management of trauma hemorrhage: A systematic review of randomized controlled trials. Crit. Care 2011, 15 (2), R92 10.1186/cc10096. 21392371 PMC3219356

[ref3] Faria I.; Thivalapill N.; Makin J.; Puyana J. C.; Raykar N. Bleeding, Hemorrhagic Shock, and the Global Blood Supply. Crit Care Clin. 2022, 38 (4), 775–793. 10.1016/j.ccc.2022.06.013. 36162910

[ref4] Montazerian H.; Davoodi E.; Baidya A.; et al. Engineered Hemostatic Biomaterials for Sealing Wounds. Chem. Rev. 2022, 122, 12864 10.1021/acs.chemrev.1c01015. 35731958

[ref5] Leitão A. F.; Faria M. A.; Faustino A. M. R.; et al. A Novel Small-Caliber Bacterial Cellulose Vascular Prosthesis: Production, Characterization, and Preliminary in Vivo Testing. Macromol Biosci. 2016, 16 (1), 139–150. 10.1002/mabi.201500251. 26388180

[ref6] Scherner M.; Reutter S.; Klemm D.; et al. In vivo application of tissue-engineered blood vessels of bacterial cellulose as small arterial substitutes: Proof of concept?. Journal of Surgical Research. 2014, 189 (2), 340–347. 10.1016/j.jss.2014.02.011. 24726059

[ref7] Wippermann J.; Schumann D.; Klemm D.; Kosmehl H.; Salehi-Gelani S.; Wahlers T. Preliminary Results of Small Arterial Substitute Performed with a New Cylindrical Biomaterial Composed of Bacterial Cellulose. European Journal of Vascular and Endovascular Surgery. 2009, 37 (5), 592–596. 10.1016/j.ejvs.2009.01.007. 19231251

[ref8] Schumann D. A.; Wippermann J.; Klemm D. O.; et al. Artificial vascular implants from bacterial cellulose: Preliminary results of small arterial substitutes. Cellulose. 2009, 16 (5), 877–885. 10.1007/s10570-008-9264-y. 19231251

[ref9] Bäckdahl H.; Esguerra M.; Delbro D.; Risberg B.; Gatenholm P. Engineering microporosity in bacterial cellulose scaffolds. J Tissue Eng Regen Med. 2008, 2 (6), 320–330. 10.1002/term.97. 18615821

[ref10] Hu Q.; Tang H.; Yao Y.; Liu S.; Zhang H.; Ramalingam M. Rapid fabrication of gelatin-based scaffolds with prevascularized channels for organ regeneration. Biomedical Materials (Bristol). 2021, 16 (4), 045010 10.1088/1748-605X/abef7b. 33730706

[ref11] Li H.; Cheng F.; Li W. Expanding sacrificially printed microfluidic channel-embedded paper devices for construction of volumetric tissue models in vitro. Biofabrication. 2020, 12 (4), 045027 10.1088/1758-5090/abb11e. 32945271 PMC7847249

[ref12] Sämfors S.; Karlsson K.; Sundberg J.; Markstedt K.; Gatenholm P. Biofabrication of bacterial nanocellulose scaffolds with complex vascular structure. Biofabrication. 2019, 11 (4), 045010 10.1088/1758-5090/ab2b4f. 31220812

[ref13] Karahaliloğlu Z.; Demirbilek M.; Ulusoy I.; Gümüşkaya B.; Baki Denkbaş E. Hemostatic activities of nano/microporous bilayer dressings in a femoral artery bleeding rat model. J. Appl. Polym. Sci. 2016, 133 (28), 43657 10.1002/app.43657.

[ref14] Karahaliloğlu Z.; Demirbilek M.; Ulusoy İ.; Gümüşkaya B.; Denkbaş E. B. Active nano/microbilayer hemostatic agents for diabetic rat bleeding model. J Biomed Mater Res B Appl Biomater. 2017, 105 (6), 1573–1585. 10.1002/jbm.b.33696. 27126123

[ref15] Cao S.; Yang Y.; Zhang S.; Liu K.; Chen J. Multifunctional dopamine modification of green antibacterial hemostatic sponge. Materials Science and Engineering C. 2021, 127, 112227 10.1016/j.msec.2021.112227. 34225872

[ref16] Yuan H.; Chen L.; Hong F. F. A Biodegradable Antibacterial Nanocomposite Based on Oxidized Bacterial Nanocellulose for Rapid Hemostasis and Wound Healing. ACS Appl Mater Interfaces. 2020, 12 (3), 3382–3392. 10.1021/acsami.9b17732. 31880915

[ref17] Revin V. V.; Liyaskina E. V.; Parchaykina M. V.; et al. Bacterial Cellulose-Based Polymer Nanocomposites: A Review. Polymers (Basel). 2022, 14 (21), 4670 10.3390/polym14214670. 36365662 PMC9654748

[ref18] Gorgieva S.; Trček J. Bacterial cellulose: Production, modification and perspectives in biomedical applications. Nanomaterials 2019, 9 (10), 1352 10.3390/nano9101352. 31547134 PMC6835293

[ref19] Mecwan M.; Li J.; Falcone N.; et al. Recent advances in biopolymer-based hemostatic materials. Regen Biomater. 2022, 9, 9 10.1093/rb/rbac063. PMC9522468 36196294

[ref20] Herliana H.; Yusuf H. Y.; Laviana A.; Wandawa G.; Cahyanto A. Characterization and Analysis of Chitosan-Gelatin Composite-Based Biomaterial Effectivity as Local Hemostatic Agent: A Systematic Review. Polymers (Basel) 2023, 15 (3), 575 10.3390/polym15030575. 36771876 PMC9920696

[ref21] Mirjalili F.; Mahmoodi M. Controlled release of protein from gelatin/chitosan hydrogel containing platelet-rich fibrin encapsulated in chitosan nanoparticles for accelerated wound healing in an animal model. Int J Biol Macromol. 2023, 225, 588–604. 10.1016/j.ijbiomac.2022.11.117. 36403766

[ref22] Wang J.; Li C.; Zhang W.; et al. A contact-polymerizable hemostatic powder for rapid hemostasis. Biomater Sci. 2023, 11, 3616 10.1039/D3BM00075C. 37010060

[ref23] Post A.; Diaz-Rodriguez P.; Balouch B.; et al. Elucidating the role of graft compliance mismatch on intimal hyperplasia using an ex vivo organ culture model. Acta Biomater. 2019, 89, 84–94. 10.1016/j.actbio.2019.03.025. 30878448 PMC6558989

[ref24] Patel D. K.; Dutta S. D.; Lim K. T. Nanocellulose-based polymer hybrids and their emerging applications in biomedical engineering and water purification. RSC Adv. 2019, 9 (33), 19143–19162. 10.1039/C9RA03261D. 35516880 PMC9065078

[ref25] Lin N.; Dufresne A. Nanocellulose in biomedicine: Current status and future prospect. Eur. Polym. J. 2014, 59, 302–325. 10.1016/j.eurpolymj.2014.07.025.

[ref26] Nicu R.; Ciolacu F.; Ciolacu D. E. Advanced functional materials based on nanocellulose for pharmaceutical/medical applications. Pharmaceutics 2021, 13 (8), 1125 10.3390/pharmaceutics13081125. 34452086 PMC8399340

[ref27] Liu L.; Ji X.; Mao L.; et al. Hierarchical-structured bacterial cellulose/potato starch tubes as potential small-diameter vascular grafts. Carbohydr. Polym. 2022, 281, 119034 10.1016/j.carbpol.2021.119034. 35074114

[ref28] Zahedmanesh H.; MacKle J. N.; Sellborn A.; et al. Bacterial cellulose as a potential vascular graft: Mechanical characterization and constitutive model development. J Biomed Mater Res B Appl Biomater. 2011, 97B (1), 105–113. 10.1002/jbm.b.31791. 21290588

[ref29] Bäckdahl H.; Helenius G.; Bodin A.; et al. Mechanical properties of bacterial cellulose and interactions with smooth muscle cells. Biomaterials. 2006, 27 (9), 2141–2149. 10.1016/j.biomaterials.2005.10.026. 16310848

[ref30] Weber C.; Reinhardt S.; Eghbalzadeh K.; et al. Patency and in vivo compatibility of bacterial nanocellulose grafts as small-diameter vascular substitute. J Vasc Surg. 2018, 68 (6), 177S–187S. 10.1016/j.jvs.2017.09.038. 29248244

[ref31] Rothe H.; Rost J.; Kramer F.; et al. Bacterial nanocellulose: Reinforcement of compressive strength using an adapted Mobile Matrix Reservoir Technology and suitable post-modification strategies. J Mech Behav Biomed Mater. 2022, 125, 104978 10.1016/j.jmbbm.2021.104978. 34837799

[ref32] Wacker M.; Riedel J.; Walles H. Comparative evaluation on impacts of fibronectin, heparin–chitosan, and albumin coating of bacterial nanocellulose small-diameter vascular grafts on endothelialization in vitro. Nanomaterials. 2021, 11 (8), 1952 10.3390/nano11081952. 34443783 PMC8398117

[ref33] Ladak S. S.; McQueen L. W.; Layton G. R.; Aujla H.; Adebayo A.; Zakkar M. The Role of Endothelial Cells in the Onset, Development and Modulation of Vein Graft Disease. Cells. 2022, 11 (19), 3066 10.3390/cells11193066. 36231026 PMC9561968

[ref34] Fink H.; Faxälv L.; Molnár G. F.; et al. Real-time measurements of coagulation on bacterial cellulose and conventional vascular graft materials. Acta Biomater. 2010, 6 (3), 1125–1130. 10.1016/j.actbio.2009.09.019. 19800035

[ref35] Tang J.; Li X.; Bao L.; Chen L.; Hong F. F. Comparison of two types of bioreactors for synthesis of bacterial nanocellulose tubes as potential medical prostheses including artificial blood vessels. J. Chem. Technol. Biotechnol. 2017, 92 (6), 1218–1228. 10.1002/jctb.5111.

[ref36] Putra A.; Kakugo A.; Furukawa H.; Gong J. P.; Osada Y. Tubular bacterial cellulose gel with oriented fibrils on the curved surface. Polymer (Guildf). 2008, 49 (7), 1885–1891. 10.1016/j.polymer.2008.02.022.

[ref37] Bao L.; Tang J.; Hong F. F.; Lu X.; Chen L. Physicochemical Properties and In Vitro Biocompatibility of Three Bacterial Nanocellulose Conduits for Blood Vessel Applications. Carbohydr. Polym. 2020, 239, 116246 10.1016/j.carbpol.2020.116246. 32414454

[ref38] Bodin A.; Bäckdahl H.; Gustafsson L.; Risberg B.; Gatenholm P. Manufacturing and Characterization of Bacterial Cellulose Tubes using Two Different Fermentation Techniques. Modern Multidisciplinary Applied Microbiology: Exploiting Microbes and Their Interactions; Wiley: 2008; pp 619–622. 10.1002/9783527611904.ch110.

[ref39] Bodin A.; Ahrenstedt L.; Fink H.; Brumer H.; Risberg B.; Gatenholm P. Modification of nanocellulose with a xyloglucan-RGD conjugate enhances adhesion and proliferation of endothelial cells: Implications for tissue engineering. Biomacromolecules. 2007, 8 (12), 3697–3704. 10.1021/bm070343q. 18031014

[ref40] Fink H.; Ahrenstedt L.; Bodin A.; et al. Bacterial cellulose modified with xyloglucan bearing the adhesion peptide RGD promotes endothelial cell adhesion and metabolism-a promising modification for vascular grafts. J Tissue Eng Regen Med. 2011, 5 (6), 454–463. 10.1002/term.334. 21604383

[ref41] Andrade F. K.; Costa R.; Domingues L.; Soares R.; Gama M. Improving bacterial cellulose for blood vessel replacement: Functionalization with a chimeric protein containing a cellulose-binding module and an adhesion peptide. Acta Biomater. 2010, 6 (10), 4034–4041. 10.1016/j.actbio.2010.04.023. 20438872

[ref42] Wang B.; Lv X.; Chen S.; et al. Bacterial cellulose/gelatin scaffold loaded with VEGF-silk fibroin nanoparticles for improving angiogenesis in tissue regeneration. Cellulose. 2017, 24 (11), 5013–5024. 10.1007/s10570-017-1472-x.

[ref43] Zhang Q.; He S.; Zhu X.; et al. Heparinization and hybridization of electrospun tubular graft for improved endothelialization and anticoagulation. Materials Science and Engineering C. 2021, 122, 111861 10.1016/j.msec.2020.111861. 33641887

[ref44] Sabino R.; Popat K. Evaluating Whole Blood Clotting in vitro on Biomaterial Surfaces. Bio Protoc. 2020, 10 (3), e3505 10.21769/BioProtoc.3505. PMC7842529 33654732

[ref45] Goyal T.; Schmotzer C. L. Validation of hemolysis index thresholds optimizes detection of clinically significant hemolysis. Am J Clin Pathol. 2015, 143 (4), 579–583. 10.1309/AJCPDUDE1HRA0YMR. 25780011

[ref46] International Organization for Standardization. Biological Evaluation of Medical Devices-Part 4: Selection of Tests for Interactions with Blood.; 2002.

[ref47] Zang S.; Zhang R.; Chen H.; et al. Investigation on artificial blood vessels prepared from bacterial cellulose. Materials Science and Engineering C. 2015, 46, 111–117. 10.1016/j.msec.2014.10.023. 25491966

[ref48] Leitão A. F.; Gupta S.; Silva J. P.; Reviakine I.; Gama M. Hemocompatibility study of a bacterial cellulose/polyvinyl alcohol nanocomposite. Colloids Surf B Biointerfaces. 2013, 111, 493–502. 10.1016/j.colsurfb.2013.06.031. 23880088

[ref49] Osorio M.; Cañas A.; Puerta J. Ex Vivo and In Vivo Biocompatibility Assessment (Blood and Tissue) of Three-Dimensional Bacterial Nanocellulose Biomaterials for Soft Tissue Implants. Sci Rep. 2019, 9 (1), 10553 10.1038/s41598-019-46918-x. 31332259 PMC6646330

[ref50] Hu Z.; Lu S.; Cheng Y. Investigation of the effects of molecular parameters on the hemostatic properties of chitosan. Molecules. 2018, 23 (12), 3147 10.3390/molecules23123147. 30513622 PMC6321099

[ref51] Hunter D. T.; Allensworth J. L. Improved coagulation screening by an activated recalcification test. J Clin Pathol. 1967, 20 (3), 244–248. 10.1136/jcp.20.3.244. 5602556 PMC473477

[ref52] Badv M.; Jaffer I. H.; Weitz J. I.; Didar T. F. An omniphobic lubricant-infused coating produced by chemical vapor deposition of hydrophobic organosilanes attenuates clotting on catheter surfaces. Sci Rep. 2017, 7 (1), 11639 10.1038/s41598-017-12149-1. 28912558 PMC5599680

[ref53] Badv M.; Alonso-Cantu C.; Shakeri A.; Hosseinidoust Z.; Weitz J. I.; Didar T. F. Biofunctional Lubricant-Infused Vascular Grafts Functionalized with Silanized Bio-Inks Suppress Thrombin Generation and Promote Endothelialization. ACS Biomater Sci Eng. 2019, 5 (12), 6485–6496. 10.1021/acsbiomaterials.9b01062. 33417801

[ref54] Badv M.; Imani S. M.; Weitz J. I.; Didar T. F. Lubricant-Infused Surfaces with Built-In Functional Biomolecules Exhibit Simultaneous Repellency and Tunable Cell Adhesion. ACS Nano. 2018, 12 (11), 10890–10902. 10.1021/acsnano.8b03938. 30352507

[ref55] Weber M.; Steinle H.; Golombek S.; et al. Blood-Contacting Biomaterials: In Vitro Evaluation of the Hemocompatibility. Front Bioeng Biotechnol. 2018, 6, 6 10.3389/fbioe.2018.00099. 30062094 PMC6054932

[ref56] Briselli M. F.; Ellman L.; Briselli M. P.; Leonard E. Kaolin-correctable Prolongation of the Activated Partial Thromboplastin Time. Am J Clin Pathol. 1980, 74, 677–680. 10.1093/ajcp/74.5.677. 7446472

[ref57] Badv M.; Bayat F.; Weitz J. I.; Didar T. F. Single and multi-functional coating strategies for enhancing the biocompatibility and tissue integration of blood-contacting medical implants. Biomaterials. 2020, 258, 120291 10.1016/j.biomaterials.2020.120291. 32798745

[ref58] Zhang S.; Li L.; Ren X.; Huang T. S. N-halamine modified multiporous bacterial cellulose with enhanced antibacterial and hemostatic properties. Int J Biol Macromol. 2020, 161, 1070–1078. 10.1016/j.ijbiomac.2020.06.053. 32531364

[ref59] Badv M.; Weitz J. I.; Didar T. F. Lubricant-Infused PET Grafts with Built-In Biofunctional Nanoprobes Attenuate Thrombin Generation and Promote Targeted Binding of Cells. Small. 2019, 15 (51), 1905562 10.1002/smll.201905562. 31773877

[ref60] Sutherland D. W.; McEleney A.; de Almeida M.; et al. Characterization of main pulmonary artery and valve annulus region of piglets using echocardiography, uniaxial tensile testing, and a novel non-destructive technique. Front Cardiovasc Med. 2022, 9, 9 10.3389/fcvm.2022.884116. PMC9459108 36093160

[ref61] Ross C.; Bird J.; Little A. Mechanics of Solids, 3rd ed.; Routledge: 2022.

[ref62] Eichhorn S. J.; Young R. J. The Young’s modulus of a microcrystalline cellulose. Cellulose. 2001, 8, 197–207. 10.1023/A:1013181804540.

[ref63] Griffin M.; Premakumar Y.; Seifalian A.; Butler P. E.; Szarko M. Biomechanical characterization of human soft tissues using indentation and tensile testing. Journal of Visualized Experiments. 2016, 2016 (118), e54872 10.3791/54872. PMC5226394 28060331

[ref64] Abbott W. M.; Megerman J.; Hasson J. E.; L’Italien G.; Warnock D. F. Effect of compliance mismatch on vascular graft patency. J Vasc Surg. 1987, 5 (2), 376–382. 10.1067/mva.1987.avs0050376. 3102762

[ref65] Zhou M.; Yu Y.; Chen R.; et al. Wall shear stress and its role in atherosclerosis. Front Cardiovasc Med. 2023, 10, 1083547 10.3389/fcvm.2023.1083547. 37077735 PMC10106633

[ref66] Bao L.; Hong F. F.; Li G.; Hu G.; Chen L. Implantation of air-dried bacterial nanocellulose conduits in a small-caliber vascular prosthesis rabbit model. Materials Science and Engineering C. 2021, 122, 111922 10.1016/j.msec.2021.111922. 33641915

[ref67] Maleki S.; Shamloo A.; Kalantarnia F. Tubular TPU/SF nanofibers covered with chitosan-based hydrogels as small-diameter vascular grafts with enhanced mechanical properties. Sci Rep. 2022, 12 (1), 6179 10.1038/s41598-022-10264-2. 35418612 PMC9008019

[ref68] Montazerian H.; Davoodi E.; Baidya A.; et al. Bio-macromolecular design roadmap towards tough bioadhesives. Chem Soc Rev. 2022, 51 (21), 9127–9173. 10.1039/D2CS00618A. 36269075 PMC9810209

[ref69] Laterreur V.; Ruel J.; Auger F. A.; et al. Comparison of the direct burst pressure and the ring tensile test methods for mechanical characterization of tissue-engineered vascular substitutes. J Mech Behav Biomed Mater. 2014, 34, 253–263. 10.1016/j.jmbbm.2014.02.017. 24631624

[ref70] Volova T. G.; Shumilova A. A.; Shidlovskiy I. P.; et al. Antibacterial properties of films of cellulose composites with silver nanoparticles and antibiotics. Polym Test. 2018, 65, 54–68. 10.1016/j.polymertesting.2017.10.023.

[ref71] Vismara E.; Bernardi A.; Bongio C. Bacterial nanocellulose and its surface modification by glycidyl methacrylate and ethylene glycol dimethacrylate. Incorporation of vancomycin and ciprofloxacin. Nanomaterials. 2019, 9 (12), 1668 10.3390/nano9121668. 31766754 PMC6955863

[ref72] Ye S.; Jiang L.; Wu J.; et al. Flexible Amoxicillin-Grafted Bacterial Cellulose Sponges for Wound Dressing: In Vitro and in Vivo Evaluation. ACS Appl Mater Interfaces. 2018, 10 (6), 5862–5870. 10.1021/acsami.7b16680. 29345902

[ref73] He W.; Huang X.; Zheng Y.; et al. In situ synthesis of bacterial cellulose/copper nanoparticles composite membranes with long-term antibacterial property. J Biomater Sci Polym Ed. 2018, 29 (17), 2137–2153. 10.1080/09205063.2018.1528518. 30280964

[ref74] Mohite B. V.; Patil S. V. In situ development of nanosilver-impregnated bacterial cellulose for sustainable released antimicrobial wound dressing. J Appl Biomater Funct Mater. 2016, 14 (1), 53–58. 10.5301/jabfm.5000257. 26689818

[ref75] Wu C. N.; Fuh S. C.; Lin S. P.; et al. TEMPO-Oxidized Bacterial Cellulose Pellicle with Silver Nanoparticles for Wound Dressing. Biomacromolecules. 2018, 19 (2), 544–554. 10.1021/acs.biomac.7b01660. 29334612

[ref76] Mohammadnejad J.; Yazdian F.; Omidi M.; Rostami A. D.; Rasekh B.; Fathinia A. Graphene oxide/silver nanohybrid: Optimization, antibacterial activity and its impregnation on bacterial cellulose as a potential wound dressing based on GO-Ag nanocomposite-coated BC. Eng Life Sci. 2018, 18 (5), 298–307. 10.1002/elsc.201700138. 32624909 PMC6999449

[ref77] Khamrai M.; Banerjee S. L.; Paul S.; Ghosh A. K.; Sarkar P.; Kundu P. P. A Mussel Mimetic, Bioadhesive, Antimicrobial Patch Based on Dopamine-Modified Bacterial Cellulose/rGO/Ag NPs: A Green Approach toward Wound-Healing Applications. ACS Sustain Chem Eng. 2019, 7 (14), 12083–12097. 10.1021/acssuschemeng.9b01163.

[ref78] Ul-Islam M.; Khan T.; Khattak W. A.; Park J. K. Bacterial cellulose-MMTs nanoreinforced composite films: Novel wound dressing material with antibacterial properties. Cellulose. 2013, 20 (2), 589–596. 10.1007/s10570-012-9849-3.

[ref79] Sajjad W.; Khan T.; Ul-Islam M.; et al. Development of modified montmorillonite-bacterial cellulose nanocomposites as a novel substitute for burn skin and tissue regeneration. Carbohydr. Polym. 2019, 206, 548–556. 10.1016/j.carbpol.2018.11.023. 30553356

[ref80] Zhang P.; Chen L.; Zhang Q.; Hong F. F. Using in situ dynamic cultures to rapidly biofabricate fabric-reinforced composites of chitosan/bacterial nanocellulose for antibacterial wound dressings. Front Microbiol. 2016, 7 (MAR), 260 10.3389/fmicb.2016.00260. 26973634 PMC4777949

[ref81] Zhao H.; Zhang L.; Zheng S.; et al. Bacteriostatic activity and cytotoxicity of bacterial cellulose-chitosan film loaded with in-situ synthesized silver nanoparticles. Carbohydr. Polym. 2022, 281, 119017 10.1016/j.carbpol.2021.119017. 35074133

[ref82] Kasapgil E.; Badv M.; Cantú C. A.; et al. Polysiloxane Nanofilaments Infused with Silicone Oil Prevent Bacterial Adhesion and Suppress Thrombosis on Intranasal Splints. ACS Biomater Sci Eng. 2021, 7 (2), 541–552. 10.1021/acsbiomaterials.0c01487. 33470781

[ref83] Bernardelli de Mattos I.; Nischwitz S. P.; Tuca A. C.; et al. Delivery of antiseptic solutions by a bacterial cellulose wound dressing: Uptake, release and antibacterial efficacy of octenidine and povidone-iodine. Burns. 2020, 46 (4), 918–927. 10.1016/j.burns.2019.10.006. 31653329

[ref84] Ghasemlou M.; Daver F.; Ivanova E. P.; Adhikari B. Bio-inspired sustainable and durable superhydrophobic materials: From nature to market. J Mater Chem A Mater. 2019, 7 (28), 16643–16670. 10.1039/C9TA05185F.

[ref85] Bonev B.; Hooper J.; Parisot J. Principles of assessing bacterial susceptibility to antibiotics using the agar diffusion method. J. Antimicrob. Chemother. 2008, 61 (6), 1295–1301. 10.1093/jac/dkn090. 18339637

[ref86] Cushnie T. P. T.; Cushnie B.; Echeverría J.; et al. Bioprospecting for Antibacterial Drugs: A Multidisciplinary Perspective on Natural Product Source Material, Bioassay Selection and Avoidable Pitfalls. Pharm. Res. 2020, 37 (7), 125 10.1007/s11095-020-02849-1. 32529587

[ref87] Parente E.; Brienza C.; Moles M.; Ricciardi A. A comparison of methods for the measurement of bacteriocin activity. J Microbiol Methods. 1995, 22, 95–108. 10.1016/0167-7012(94)00068-I.

[ref88] Balouiri M.; Sadiki M.; Ibnsouda S. K. Methods for in vitro evaluating antimicrobial activity: A review. J Pharm Anal. 2016, 6 (2), 71–79. 10.1016/j.jpha.2015.11.005. 29403965 PMC5762448

[ref89] Li Y.; Zha X.; Xiong X.; et al. A promising wound dressing from regenerated silk fibroin sponge with sustain-ed release of silver nanoparticles. J Renew Mater. 2021, 9 (2), 295–310. 10.32604/jrm.2021.012271.

[ref90] Millette M.; Luquet F. M.; Lacroix M. In vitro growth control of selected pathogens by Lactobacillus acidophilus- and Lactobacillus casei-fermented milk. Lett Appl Microbiol. 2007, 44 (3), 314–319. 10.1111/j.1472-765X.2006.02060.x. 17309510

[ref91] Perez-Gavilan A.; de Castro J. V.; Arana A. Antibacterial activity testing methods for hydrophobic patterned surfaces. Sci Rep. 2021, 11 (1), 6675 10.1038/s41598-021-85995-9. 33758227 PMC7988007

[ref92] Mann E. E.; Manna D.; Mettetal M. R. Surface micropattern limits bacterial contamination. Antimicrob Resist Infect Control. 2014, 3 (1), 28 10.1186/2047-2994-3-28. 25232470 PMC4166016

[ref93] Bi Y.; Xia G.; Shi C.; et al. Therapeutic strategies against bacterial biofilms. Fundamental Research. 2021, 1 (2), 193–212. 10.1016/j.fmre.2021.02.003.

[ref94] Villegas M.; Zhang Y.; Badv M. Enhancing osseointegration and mitigating bacterial biofilms on medical-grade titanium with chitosan-conjugated liquid-infused coatings. Sci Rep. 2022, 12 (1), 5380 10.1038/s41598-022-09378-4. 35354896 PMC8967836

[ref95] Mack D.; Nedelmann M.; Krokotsch A.; Schwarzkopf A.; Heesemann J.; Laufs’ R. Characterization of Transposon Mutants of Biofilm-Producing Staphylococcus epidermidis Impaired in the Accumulative Phase of Biofilm Production: Genetic Identification of a Hexosamine-Containing Polysaccharide Intercellular Adhesin. Infection and Immunology. 1994, 62 (8), 3244–3253. 10.1128/iai.62.8.3244-3253.1994. PMC302952 8039894

[ref96] O’Toole G.; Kaplan H. B.; Kolter R. Biofilm Formation as Microbial Development. Annu. Rev. Microbiol. 2000, 54 (1), 49–79. 10.1146/annurev.micro.54.1.49. 11018124

[ref97] Merritt J. H.; Kadouri D. E.; O’Toole G. A. Growing and Analyzing Static Biofilms. Curr Protoc Microbiol. 2005, 00, 1B.1.1–1B.1.17. 10.1002/9780471729259.mc01b01s00. PMC4568995 18770545

[ref98] Wilson C.; Lukowicz R.; Merchant S.; Quantitative and Qualitative Assessment Methods for Biofilm Growth: A Mini-Review. Res. Rev. J. Eng. Technol. 2017; Vol 6; http://www.rroij.com/open-access/quantitative-and-qualitative-assessment-methods-for-biofilm-growth-a-minireview-.pdf. PMC6133255 30214915

[ref99] Guzmán-Soto I.; Mctiernan C.; Gonzalez-Gomez M.; et al. Mimicking biofilm formation and development: Recent progress in in vitro and in vivo biofilm models. iScience. 2021, 24, 102443 10.1016/j.isci.2021.102443. 34013169 PMC8113887

[ref100] Esguerra M.; Fink H.; Laschke M. W.; et al. Intravital fluorescent microscopic evaluation of bacterial cellulose as scaffold for vascular grafts. J Biomed Mater Res A. 2010, 93A (1), 140–149. 10.1002/jbm.a.32516. 19536832

[ref101] Helenius G.; Bäckdahl H.; Bodin A.; Nannmark U.; Gatenholm P.; Risberg B. In vivo biocompatibility of bacterial cellulose. J Biomed Mater Res A. 2006, 76A (2), 431–438. 10.1002/jbm.a.30570. 16278860

[ref102] Hu G.; Chen L.; Zhao S.; Hong F. F. Mercerization of tubular bacterial nanocellulose for control of the size and performance of small-caliber vascular grafts. Chemical Engineering Journal. 2022, 428, 131104 10.1016/j.cej.2021.131104.

[ref103] Piasecka-Zelga J.; Zelga P.; Gronkowska K.; et al. Toxicological and Sensitization Studies of Novel Vascular Prostheses Made of Bacterial Nanocellulose Modified with Chitosan (MBC) for Application as the Tissue-Engineered Blood Vessels. Regen Eng Transl Med. 2021, 7 (2), 218–233. 10.1007/s40883-021-00209-y.

[ref104] Bäckdahl H.; Risberg B.; Gatenholm P. Observations on bacterial cellulose tube formation for application as vascular graft. Materials Science and Engineering C. 2011, 31 (1), 14–21. 10.1016/j.msec.2010.07.010.

[ref105] Bao L.; Li C.; Tang M.; Chen L.; Hong F. F. Potential of a Composite Conduit with Bacterial Nanocellulose and Fish Gelatin for Application as Small-Diameter Artificial Blood Vessel. Polymers (Basel). 2022, 14 (20), 4367 10.3390/polym14204367. 36297946 PMC9610583

[ref106] Zhang S.; Li J.; Chen S.; Zhang X.; Ma J.; He J. Oxidized cellulose-based hemostatic materials. Carbohydr. Polym. 2020, 230, 115585 10.1016/j.carbpol.2019.115585. 31887971

[ref107] Queirós E. C.; Pinheiro S. P.; Pereira J. E.; et al. Hemostatic Dressings Made of Oxidized Bacterial Nanocellulose Membranes. Polysaccharides. 2021, 2 (1), 80–99. 10.3390/polysaccharides2010006.

[ref108] Křížová P.; Mášová L.; Suttnar J.; et al. The influence of intrinsic coagulation pathway on blood platelets activation by oxidized cellulose. J Biomed Mater Res A. 2007, 82A (2), 274–280. 10.1002/jbm.a.31060. 17274026

[ref109] Ryšavá J.; Dyr J. E.; Homola J.; et al. Surface interactions of oxidized cellulose with fibrin(ogen) and blood platelets. Sensors and Actuators, B: Chemical. 2003, 90, 243–249. 10.1016/S0925-4005(03)00035-2.

[ref110] Bian J.; Bao L.; Gao X. Bacteria-engineered porous sponge for hemostasis and vascularization. J Nanobiotechnology. 2022, 20 (1), 47 10.1186/s12951-022-01254-7. 35062972 PMC8780714

[ref111] Yu P.; Zhong W. Hemostatic materials in wound care. Burns Trauma. 2021, 9, 9 10.1093/burnst/tkab019. PMC8445204 34541007

[ref112] Manon-Jensen T.; Kjeld N. G.; Karsdal M. A. Collagen-mediated hemostasis. Journal of Thrombosis and Haemostasis. 2016, 14 (3), 438–448. 10.1111/jth.13249. 26749406

[ref113] Wang Y. W.; Liu C. C.; Cherng J. H. Biological effects of chitosan-based dressing on hemostasis mechanism. Polymers (Basel). 2019, 11 (11), 1906 10.3390/polym11111906. 31752424 PMC6918334

[ref114] Khan M. A.; Mujahid M. A review on recent advances in chitosan based composite for hemostatic dressings. Int J Biol Macromol. 2019, 124, 138–147. 10.1016/j.ijbiomac.2018.11.045. 30447365

[ref115] Wei W.; Liu J.; Peng Z.; Liang M.; Wang Y.; Wang X. Gellable silk fibroin-polyethylene sponge for hemostasis. Artif Cells Nanomed Biotechnol. 2020, 48 (1), 28–36. 10.1080/21691401.2019.1699805. 31852256

[ref116] Sultan M. T.; Hong H.; Lee O. J. Silk Fibroin-Based Biomaterials for Hemostatic Applications. Biomolecules. 2022, 12 (5), 660 10.3390/biom12050660. 35625588 PMC9138874

[ref117] Boer C.; Meesters M. I.; Veerhoek D.; Vonk A. B. A. Anticoagulant and side-effects of protamine in cardiac surgery: a narrative review. Br J Anaesth. 2018, 120 (5), 914–927. 10.1016/j.bja.2018.01.023. 29661409

[ref118] Shahriari-Khalaji M.; Hu G.; Chen L.; et al. Functionalization of Aminoalkylsilane-Grafted Bacterial Nanocellulose with ZnO-NPs-Doped Pullulan Electrospun Nanofibers for Multifunctional Wound Dressing. ACS Biomater Sci Eng. 2021, 7 (8), 3933–3946. 10.1021/acsbiomaterials.1c00444. 34296596

[ref119] Bin-Jardan L. I.; Almadani D. I.; Almutairi L. S.; et al. Inorganic Compounds as Remineralizing Fillers in Dental Restorative Materials: Narrative Review. Int J Mol Sci. 2023, 24 (9), 8295 10.3390/ijms24098295. 37176004 PMC10179470

[ref120] Awad M. E.; López-Galindo A.; Setti M.; El-Rahmany M. M.; Iborra C. V. Kaolinite in pharmaceutics and biomedicine. Int. J. Pharm. 2017, 533 (1), 34–48. 10.1016/j.ijpharm.2017.09.056. 28943206

[ref121] Sai S.; Kondapalli A.; Czyz C. N.; Stacey A. W.; Cahill K V; Foster J. A. Use of Kaolin-impregnated Gauze for Improvement of Intraoperative Hemostasis and Postoperative Wound Healing in Blepharoplasty. Clinical and Aesthetic Dermatology. 2016, 9 (6), 51–55. PMC4928457 27386052

[ref122] Li G.; Quan K.; Liang Y.; et al. Graphene-Montmorillonite Composite Sponge for Safe and Effective Hemostasis. ACS Appl Mater Interfaces. 2016, 8 (51), 35071–35080. 10.1021/acsami.6b13302. 27935296

[ref123] Yang Z.; Ye T.; Ma F. Preparation of Chitosan/Clay Composites for Safe and Effective Hemorrhage Control. Molecules. 2022, 27 (8), 2571 10.3390/molecules27082571. 35458768 PMC9026824

[ref124] Wanna D.; Alam C.; Toivola D. M.; Alam P. Bacterial cellulose-kaolin nanocomposites for application as biomedical wound healing materials. Advances in Natural Sciences: Nanoscience and Nanotechnology. 2013, 4 (4), 045002 10.1088/2043-6262/4/4/045002.

[ref125] Véliz D. S.; Alam C.; Toivola D. M.; Toivakka M.; Alam P. On the non-linear attachment characteristics of blood to bacterial cellulose/kaolin biomaterials. Colloids Surf B Biointerfaces. 2014, 116, 176–182. 10.1016/j.colsurfb.2013.12.038. 24457185

[ref126] Tsuda T.; Yoshimura H.; Hamasaki N. Effect of phosphatidylcholine, phosphatidylethanolamine and lysophosphatidylcholine on the protein C/protein S anticoagulation system. Blood Coagulation and Fibrinolysis. 2006, 17, 453–458. 10.1097/01.mbc.0000240917.71144.7b. 16905948

[ref127] Zareh M.; Davis A.; Henderson S. Reversal of warfarin-induced hemorrhage in the emergency department. Western Journal of Emergency Medicine. 2011, 12 (4), 386–392. 10.5811/westjem.2011.3.2051. 22224125 PMC3236169

[ref128] Adefisoye M. A.; Olaniran A. O. Does Chlorination Promote Antimicrobial Resistance in Waterborne Pathogens? Mechanistic Insight into Co-Resistance and Its Implication for Public Health. Antibiotics. 2022, 11 (5), 564 10.3390/antibiotics11050564. 35625208 PMC9137585

[ref129] Zhang S.; Ding F.; Liu Y.; Ren X. Glucose-responsive biomimetic nanoreactor in bacterial cellulose hydrogel for antibacterial and hemostatic therapies. Carbohydr. Polym. 2022, 292, 119615 10.1016/j.carbpol.2022.119615. 35725195

[ref130] Vielreicher M.; Kralisch D.; Völkl S.; Sternal F.; Arkudas A.; Friedrich O. Bacterial nanocellulose stimulates mesenchymal stem cell expansion and formation of stable collagen-I networks as a novel biomaterial in tissue engineering. Sci Rep. 2018, 8 (1), 9401 10.1038/s41598-018-27760-z. 29925980 PMC6010428

[ref131] Fusco D.; Meissner F.; Podesser B. K.; et al. Small-diameter bacterial cellulose-based vascular grafts for coronary artery bypass grafting in a pig model. Front Cardiovasc Med. 2022, 9, 9 10.3389/fcvm.2022.881557. PMC9548626 36225961

[ref132] Pernagallo S.; Tura O.; Wu M.; et al. Novel biopolymers to enhance endothelialisation of intra-vascular devices. Adv Healthc Mater. 2012, 1 (5), 646–656. 10.1002/adhm.201200130. 23184801

[ref133] Piterina A v.; Cloonan A. J.; Meaney C. L.; et al. ECM-based materials in cardiovascular applications: Inherent healing potential and augmentation of native regenerative processes. Int J Mol Sci. 2009, 10 (10), 4375–4417. 10.3390/ijms10104375. 20057951 PMC2790114

[ref134] Fink H.; Hong J.; Drotz K.; Risberg B.; Sanchez J.; Sellborn A. An in vitro study of blood compatibility of vascular grafts made of bacterial cellulose in comparison with conventionally-used graft materials. J Biomed Mater Res A. 2011, 97A (1), 52–58. 10.1002/jbm.a.33031. 21308986

[ref135] Li X.; Tang J.; Bao L.; Chen L.; Hong F. F. Performance improvements of the BNC tubes from unique double-silicone-tube bioreactors by introducing chitosan and heparin for application as small-diameter artificial blood vessels. Carbohydr. Polym. 2017, 178, 394–405. 10.1016/j.carbpol.2017.08.120. 29050610

[ref136] Bao L.; Hong F. F.; Li G.; Hu G.; Chen L. Improved Performance of Bacterial Nanocellulose Conduits by the Introduction of Silk Fibroin Nanoparticles and Heparin for Small-Caliber Vascular Graft Applications. Biomacromolecules. 2021, 22 (2), 353–364. 10.1021/acs.biomac.0c01211. 33290651

[ref137] Wang J.; Wan Y.; Huang Y. Immobilisation of heparin on bacterial cellulose-chitosan nano-fibres surfaces via the cross-linking technique. IET Nanobiotechnol. 2012, 6 (2), 52–57. 10.1049/iet-nbt.2011.0038. 22559707

[ref138] Andrade F. K.; Alexandre N.; Amorim I.; et al. Studies on the biocompatibility of bacterial cellulose. J Bioact Compat Polym. 2013, 28 (1), 97–112. 10.1177/0883911512467643.

[ref139] Andrade F. K.; Moreira S. M. G.; Domingues L.; Gama F. M. P. Improving the affinity of fibroblasts for bacterial cellulose using carbohydrate-binding modules fused to RGD. J Biomed Mater Res A. 2010, 92A (1), 9–17. 10.1002/jbm.a.32284. 19165790

[ref140] Weishaupt R.; Zünd J. N.; Heuberger L. Antibacterial, Cytocompatible, Sustainably Sourced: Cellulose Membranes with Bifunctional Peptides for Advanced Wound Dressings. Adv Healthc Mater. 2020, 9 (7), 1901850 10.1002/adhm.201901850. 32159927

[ref141] Kuzmenko V.; Sämfors S.; Hägg D.; Gatenholm P. Universal method for protein bioconjugation with nanocellulose scaffolds for increased cell adhesion. Materials Science and Engineering C. 2013, 33 (8), 4599–4607. 10.1016/j.msec.2013.07.031. 24094166

[ref142] Zhang L.; Wei F.; Bai Q.; et al. Oscillating Magnetic Field Regulates Cell Adherence and Endothelialization Based on Magnetic Nanoparticle-Modified Bacterial Cellulose. ACS Appl Mater Interfaces. 2020, 12 (47), 52467–52478. 10.1021/acsami.0c17213. 33170636

[ref143] Pavón J. J.; Allain J. P.; Verma D. In situ Study Unravels Bio-Nanomechanical Behavior in a Magnetic Bacterial Nano-cellulose (MBNC) Hydrogel for Neuro-Endovascular Reconstruction. Macromol Biosci. 2019, 19 (2), 1800225 10.1002/mabi.201800225. 30451373

[ref144] Arias S. L.; Shetty A.; Devorkin J.; Allain J. P. Magnetic targeting of smooth muscle cells in vitro using a magnetic bacterial cellulose to improve cell retention in tissue-engineering vascular grafts. Acta Biomater. 2018, 77, 172–181. 10.1016/j.actbio.2018.07.013. 30004023

[ref145] Osorio M.; Ortiz I.; Gañán P.; et al. Novel surface modification of three-dimensional bacterial nanocellulose with cell-derived adhesion proteins for soft tissue engineering. Materials Science and Engineering C. 2019, 100, 697–705. 10.1016/j.msec.2019.03.045. 30948106

[ref146] Palta S.; Saroa R.; Palta A. Overview of the coagulation system. Indian J Anaesth. 2014, 58 (5), 515–523. 10.4103/0019-5049.144643. 25535411 PMC4260295

[ref147] Gospodarowicz D.; Cheng J. Heparin protects basic and acidic FGF from inactivation. J Cell Physiol. 1986, 128 (3), 475–484. 10.1002/jcp.1041280317. 3528177

[ref148] Schreiber A. B.; Kenney J.; Kowalski W. J.; Frieselt R.; Mehlmant T.; Maciagt T. Interaction of endothelial cell growth factor with heparin: Characterization by receptor and antibody recognition (polypeptide hormones/endothelial cell growth factor receptor/glycosaminoglycans). Cell Biology. 1985, 82, 6138–6142. 10.1073/pnas.82.18.6138. PMC391007 2412230

[ref149] Thornton S. C.; Mueller S. N.; Levine E. M. Human Endothelial Cells: Use of Heparin in Cloning and Long-Term Serial Cultivation. American Association for Advancement of Science. 1983, 222 (4624), 623–625. 10.1126/science.6635659. 6635659

[ref150] Liu X. C.; Zhao J.; Wang Y.; Liu T. J.; Lü F.; He G. W. Heparin- and basic fibroblast growth factor-incorporated stent: A new promising method for myocardial revascularization. Journal of Surgical Research. 2010, 164 (2), 204–213. 10.1016/j.jss.2009.05.005. 19691971

[ref151] Yao Y.; Wang J.; Cui Y.; et al. Effect of sustained heparin release from PCL/chitosan hybrid small-diameter vascular grafts on anti-thrombogenic property and endothelialization. Acta Biomater. 2014, 10 (6), 2739–2749. 10.1016/j.actbio.2014.02.042. 24602806

[ref152] Haudenschild C. Morphology of intimal hyperpasia. J Vasc Surg. 1989, 10 (5), 591–592. 10.1016/0741-5214(89)90157-2.

[ref153] Conn G.; Kidane A. G.; Punshon G.; Kannan R. Y.; Hamilton G.; Seifalian A. M. Is there an alternative to systemic anticoagulation, as related to interventional biomedical devices?. Expert Rev Med Devices. 2006, 3 (2), 245–261. 10.1586/17434440.3.2.245. 16515390

[ref154] Gbyli R.; Mercaldi A.; Sundaram H.; Amoako K. A. Achieving Totally Local Anticoagulation on Blood Contacting Devices. Adv Mater Interfaces. 2018, 5 (4), 1700954 10.1002/admi.201700954.

[ref155] Linder M.; Teeri T. T. The cellulose-binding domain of the major cellobiohydrolase of Trichoderma reesei exhibits true reversibility and a high exchange rate on crystalline cellulose (cellulase/cellobiohydrolase I/protein adsorption/microcrystalline cellulose). Biochemistry. 1996, 93, 12251–12255. 10.1073/pnas.93.22.12251. PMC37976 8901566

[ref156] Levy I.; Shoseyov O. Cellulose-binding domains Biotechnological applications. Biotechnol Adv. 2002, 20, 191–213. 10.1016/S0734-9750(02)00006-X. 14550028

[ref157] Pértile R.; Moreira S.; Andrade F.; Domingues L.; Gama M. Bacterial cellulose modified using recombinant proteins to improve neuronal and mesenchymal cell adhesion. Biotechnol. Prog. 2012, 28 (2), 526–532. 10.1002/btpr.1501. 22271600

[ref158] Xing H.; Lee H.; Luo L.; Kyriakides T. R. Extracellular matrix-derived biomaterials in engineering cell function. Biotechnol Adv. 2020, 42, 107421 10.1016/j.biotechadv.2019.107421. 31381963 PMC6995418

[ref159] Osorio M.; Cañas A.; Puerta J. Ex Vivo and In Vivo Biocompatibility Assessment (Blood and Tissue) of Three-Dimensional Bacterial Nanocellulose Biomaterials for Soft Tissue Implants. Sci Rep. 2019, 9 (1), 10553 10.1038/s41598-019-46918-x. 31332259 PMC6646330

[ref160] Wan Y.; Yang S.; Peng M.; et al. Controllable synthesis of biomimetic nano/submicro-fibrous tubes for potential small-diameter vascular grafts. J Mater Chem B. 2020, 8 (26), 5694–5706. 10.1039/D0TB01002B. 32510089

[ref161] Li Y.; Jiang K.; Feng J. Construction of Small-Diameter Vascular Graft by Shape-Memory and Self-Rolling Bacterial Cellulose Membrane. Adv Healthc Mater. 2017, 6 (11), 1601343 10.1002/adhm.201601343. 28306221

[ref162] Echeverry-Rendon M.; Reece L. M.; Pastrana F. Bacterial Nanocellulose Magnetically Functionalized for Neuro-Endovascular Treatment. Macromol Biosci. 2017, 17 (6), 1600382 10.1002/mabi.201600382. 28116837

[ref163] Vijayaratnam P. R. S.; Barber T. J.; Reizes J. A. The localized hemodynamics of drug-eluting stents are not improved by the presence of magnetic struts. J Biomech Eng. 2017, 139 (1), BIO-16-1150 10.1115/1.4035263. 27893059

[ref164] Belousov A. N. Investigation of the Effect of Magnetite Nanoparticles (MCS-B) on Human Platelet Aggregation. Iberoamerican Journal of Medicine. 2020, 2, 24–29. 10.53986/ibjm.2020.0006.

[ref165] Camasão D. B.; Mantovani D. The mechanical characterization of blood vessels and their substitutes in the continuous quest for physiological-relevant performances. A critical review. Mater Today Bio. 2021, 10, 100106 10.1016/j.mtbio.2021.100106. PMC8050780 33889837

[ref166] Tang J.; Bao L.; Li X.; Chen L.; Hong F. F. Potential of PVA-doped bacterial nano-cellulose tubular composites for artificial blood vessels. J Mater Chem B. 2015, 3 (43), 8537–8547. 10.1039/C5TB01144B. 32262694

[ref167] Klemm D.; Petzold-Welcke K.; Kramer F. Biotech nanocellulose: A review on progress in product design and today’s state of technical and medical applications. Carbohydr. Polym. 2021, 254, 117313 10.1016/j.carbpol.2020.117313. 33357876

[ref168] Fried W.; Klemm D.; Kopsch V.; Koth D.; Kramer F.; Moritz S. Process for the production of hollow bodies from Microbial cellulose; WO/2013/113675; 2013. https://patentscope.wipo.int/search/en/detail.jsf?docId=WO2013113675.

[ref169] Wacker M.; Kießwetter V.; Slottosch I. In vitro hemo- And cytocompatibility of bacterial nanocelluose small diameter vascular grafts: Impact of fabrication and surface characteristics. PLoS One. 2020, 15, e0235168 10.1371/journal.pone.0235168. 32579611 PMC7313737

[ref170] Corzo Salinas D. R.; Sordelli A.; Martinez L. A.; Villoldo G.; Bernal C.; Perez M. S.; Cerrutti P.; Foresti M. L.; et al. Production of bacterial cellulose tubes for biomedical applications: Analysis of the effect of fermentation time on selected properties. Int J Biol Macromol. 2021, 189, 1–10. 10.1016/j.ijbiomac.2021.08.011. 34364942

[ref171] Klemm D.; Marsch S.; Schumann D.; Udhardt U. Method and device for producing shaped microbial cellulose for use as biomaterial, especially for microsurgery. WO2001061026A1; 2001. https://patents.google.com/patent/WO2001061026A1/de.

[ref172] Fried W.; Klemm D.; Kopsch V.; Koth D.; Kramer F.; Moritz S.; Richter T.; Schumann D.; Udhardt U. Process for Producing Hollow Bodies from Microbial Cellulose. WO2013113675A1; 2013. https://patents.google.com/patent/WO2013113675A1/en.

[ref173] Bertholdt G. Process for the production of a long hollow cellulose body. WO2007093445A1; 2007. https://patents.google.com/patent/WO2007093445A1/en.

[ref174] Klemm D.; Schumann D.; Udhardt U.; Marsch S. Bacterial synthesized cellulose- artificial blood vessels for microsurgery. Prog. Polym. Sci. 2001, 26, 1561–1603. 10.1016/S0079-6700(01)00021-1.

[ref175] Hong F.; Wei B.; Chen L. Preliminary study on biosynthesis of bacterial nanocellulose tubes in a novel double-silicone-tube bioreactor for potential vascular prosthesis. Biomed Res Int. 2015, 2015, 560365 10.1155/2015/560365. 26090420 PMC4452228

[ref176] Bodin A.; Bäckdahl H.; Fink H.; Gustafsson L.; Risberg B.; Gatenholm P. Influence of cultivation conditions on mechanical and morphological properties of bacterial cellulose tubes. Biotechnol. Bioeng. 2007, 97 (2), 425–434. 10.1002/bit.21314. 17195972

[ref177] Shin S.; Kwak H.; Shin D.; Hyun J. Solid matrix-assisted printing for three-dimensional structuring of a viscoelastic medium surface. Nat Commun. 2019, 10 (1), 4650 10.1038/s41467-019-12585-9. 31604956 PMC6789121

[ref178] Wang B.; Lv X.; Li Z.; et al. Urethra-inspired biomimetic scaffold: A therapeutic strategy to promote angiogenesis for urethral regeneration in a rabbit model. Acta Biomater. 2020, 102, 247–258. 10.1016/j.actbio.2019.11.026. 31734410

[ref179] Favi P.; Benson R.; Dhar M.; Neilsen N. Nanofiber Oriented Bacterial Cellulose Tubular Scaffolds: Mechanical Properties and Cellular Response. 5th International Conference on Nanotechnology: Fundamentals and Applications; 2014; 1–7.

[ref180] Shiru J.; Weihua T.; Hongjiang Y.; Yuanyuan J.; Huixia Z. Preparation and characterization of bacterial cellulose tube. 3rd International Conference on Bioinformatics and Biomedical Engineering; ICBBE: 2009. 10.1109/ICBBE.2009.5163226.

[ref181] Stumpf T. R.; Tang L.; Kirkwood K.; Yang X.; Zhang J.; Cao X. Production and evaluation of biosynthesized cellulose tubes as promising nerve guides for spinal cord injury treatment. J Biomed Mater Res A. 2020, 108 (6), 1380–1389. 10.1002/jbm.a.36909. 32105397

[ref182] Bodin A.; Bharadwaj S.; Wu S.; Gatenholm P.; Atala A.; Zhang Y. Tissue-engineered conduit using urine-derived stem cells seeded bacterial cellulose polymer in urinary reconstruction and diversion. Biomaterials. 2010, 31 (34), 8889–8901. 10.1016/j.biomaterials.2010.07.108. 20800278

[ref183] Orelma H.; Morales L. O.; Johansson L. S.; et al. Affibody conjugation onto bacterial cellulose tubes and bioseparation of human serum albumin. RSC Adv. 2014, 4 (93), 51440–51450. 10.1039/C4RA08882D.

[ref184] Ciechariska D.; Wietecha J.; Kazmierczak D.; Kazmierczak J. Biosynthesis of Modified Bacterial Cellulose in a Tubular Form. Fibres and Textiles in Eastern Europe 2010, 18, 98–104.

